# Odorant Binding Proteins and Chemosensory Proteins in *Episyrphus balteatus* (Diptera: Syrphidae): Molecular Cloning, Expression Profiling, and Gene Evolution

**DOI:** 10.1093/jisesa/ieaa065

**Published:** 2020-08-08

**Authors:** Hui-Ru Jia, Lin-Lin Niu, Yu-Feng Sun, Yong-Qiang Liu, Kong-Ming Wu

**Affiliations:** 1 State Key Laboratory for Biology of Plant Diseases and Insect Pests, Institute of Plant Protection, Chinese Academy of Agricultural Sciences, Beijing, China; 2 Laboratory of Agro-products Quality Safety Risk Assessment (Beijing), Institute of Food Science and Technology, Chinese Academy of Agricultural Sciences, Beijing, China

**Keywords:** *Episyrphus balteatus*, odorant-binding protein (OBP), chemosensory protein (CSP), motif-pattern, tissue expression profile

## Abstract

Aphidophagous syrphids (Diptera: Syrphidae) are important insects in agroecosystems for pollination and biological control. Insect chemoreception is essential for these processes and for insect survival and reproduction; however, molecular determinants is not well understood for these beneficial insects. Here, we used recent transcriptome data for the common hoverfly, *Episyrphus balteatus*, to characterize key molecular components of chemoreception: odorant-binding proteins (OBPs) and chemosensory proteins (CSPs). Six EbalCSPs and 44 EbalOBPs were cloned from this species, and sequence analysis showed that most share the characteristic hallmarks of their protein family, including a signal peptide and conserved cysteine signature. Some regular patterns and key conserved motifs of OBPs and CSPs in Diptera were identified using the online tool MEME. Motifs were also compared among the three OBP subgroups. Quantitative real-time PCR (qRT-PCR) showed that most of these chemosensory genes were expressed in chemosensory organs, suggesting these genes have chemoreceptive functions. An overall comparison of the Ka/Ks values of orthologous genes in *E. balteatus* and another predatory hoverfly species to analyze the evolution of these olfactory genes showed that OBPs and CSPs are under strong purifying selection. Overall, our results provide a molecular basis for further exploring the chemosensory mechanisms of *E. balteatus*, and consequently, may help us to understand the tritrophic interactions among plants, herbivorous insects, and natural enemies.

Aphidophagous syrphids are an economically important insect group, whose larvae act as efficient control agents of crop aphids, and the adults are well-known pollinators of many plant species (Chambers and Adams 1986, Smith and Chaney 2007, Ssymank et al. 2008, Hopper et al. 2011, Jauker et al. 2012, Rader et al. 2016). Like other enemies of insect pests, aphidophagous hoverflies can use a range of environmental cues such as prey-derived volatiles [(E)-β-farnesene], herbivore-induced plant volatiles (monoterpenes and sesquiterpenes), or naturally occurring general leaf volatiles (GLVs; alcohols, aldehydes, and esters) to locate their prey and oviposition sites, where these processes rely heavily on their chemosensory systems (Turlings and Tumlinson 1992; Pare and Tumlinson 1997; Sadeghi and Gilbert 2000a, 2000b; Francis et al. 2005; Harmel et al. 2007; Verheggen et al. 2008). In this context, a detailed study of the chemoreception in the hoverflies would help us to understand the plant–herbivore–natural enemy interactions and thereby maximize their function as natural enemies and pollinators.

In insects, two groups of polypeptides are commonly known to involve the process of the chemoreception: membrane-bound receptors (olfactory receptors [ORs], gustatory receptors [GRs], and ionotropic receptors [IRs]) (Buck and Axel 1991, Clyne et al. 1999, Carraher et al. 2015, He et al. 2018) and soluble binding proteins (odorant-binding proteins [OBPs] and chemosensory proteins [CSPs]) (Pelosi et al. 1981; Vogt and Riddiford 1981; Pelosi et al. 1982; He et al. 2019a, 2019b). The latter are highly concentrated in the olfactory organs and have long been considered to participate the first step of chemical communication, acting to bind and solubilize odorant molecules across the aqueous lymph of the sensilla toward the corresponding membrane-bound receptors (Calvello et al. 2003, Honson et al. 2005, Pelosi et al. 2006, Sachse and Krieger 2011, Pelosi et al. 2014).

Insect OBPs display a typical conserved cysteine pattern, and based on the number of cysteines present, they can be further divided into five distinct subgroups: classical OBPs (six conserved cysteines), minus-C OBP (four conserved cysteines), plus-C OBP (eight conserved cysteines), atypical OBP (9–10 conserved cysteines and a long C-terminus), and dimer OBP (12 conserved cysteines) (Xu et al. 2003, Zhou et al. 2004, Pelosi et al. 2006, Zhou et al. 2010). Since the first OBPs were discovered in 1981 from the giant moth *Antheraea polyphemus* (Vogt and Riddiford 1981), they have been identified and characterized in numerous insect species across multiple orders using transcriptome and genome sequencing (Zhou et al. 2006, Pelosi et al. 2014). Their indispensable role in insect chemoreception has also been demonstrated in vivo and in vitro for several species (e.g., Xu et al. 2005; Matsuo et al. 2007; Swarup et al. 2011; Siciliano et al. 2014; Wu et al. 2016; Zhu et al. 2016; He et al. 2017, 2019a).

The chemosensory proteins (CSPs) were first identified in *Drosophila melanogaster* and have since been characterized from insects in a wide range of orders, and variously called olfactory specific-D-like (OS-D-like) proteins (McKenna et al. 1994), A-10 proteins (Pikielny et al. 1994), or sensory appendage proteins (SAPs) (Robertson et al. 1999). Compared with OBPs, the proteins in this family are much smaller (around 120 amino acids), have only four conserved cysteines, and share a higher amino acid identity across insect species (Pelosi et al. 2006, 2018). As another class of small soluble binding proteins, growing experimental evidence has demonstrated that the role of some members of this family is not confined to chemical perception and may, in fact, participate in other physiological functions such as growth and development, immune response, dietary (for a review, see Pelosi et al. 2018).

As an effort to better understand the molecular basis of syrphid olfaction, in the current study, we focused on studying the two classes of odorant carrier proteins, OBPs and CSPs, in the hoverfly, *Episyrphus balteatus* (Diptera: Syrphidae), a relatively well-known predatory hoverflies worldwide. We first tested the validity of the putative 49 OBPs and six CSPs genes obtained from previously reported *E. balteatus* antennal transcriptome data (Wang et al. 2017a), by molecular cloning and sequencing, then systematically characterized the encoding genes with respect to biochemical characteristics, phylogenetic analysis, conserved cysteines, and motif patterns. We also analyzed expression patterns of these genes in different body parts using quantitative real-time PCR (qRT-PCR) and the molecular evolution of these genes in this species in comparison to another predatory hoverfly species by calculating the rate of sequence evolution (nonsynonymous to synonymous changes [Ka/Ks]).

## Materials and Methods

### Insect and Tissue Collection

Adult individuals of *E. balteatus* used in this study were originally caught in cotton fields at the Xinxiang Experimental Station of Chinese Academy of Agricultural Sciences, Henan Province, China (35.18°N, 113.52°E) in September 2017, and were then reared in our laboratory on an artificial diet at 23 ± 1°C, 65 ± 5% relative humidity (RH), and a 14:10 (L:D) h photoperiod (Jia et al. 2019).

Various tissues, including 300 antennae, 50 heads (excluding antennae), 30 thoraxes, 20 abdomens, and 100 legs of mixed sexes, were separated from newly emerged adults (<24 h old) on ice under a microscope and immediately stored at −80°C for further processing. All treatments were performed three times.

### RNA Isolation and cDNA Synthesis

Total RNA was extracted from each specimen using the Trizol Reagent (Ambion, Life Technologies, Carlsbad, CA) along with the recommended protocols. RNA sample purity and concentration were determined using a spectrophotometer (NanoDrop-2000, Thermo Scientific, Wilmington, DE). Only RNA preparations with an A260/A280 ratio between 1.9 and 2.05, and A260/A230 ratio > 1.8 were used in the following experiments.

For each sample, cDNA was synthesized from 1 µg of RNA using the FastQuant RT kit with gDNA Eraser (TianGen, Beijing, China) following the manufacturer’s instructions, in a 20 μl final volume. The cDNA was diluted to 200 ng/µl with nuclease-free water to be used in the further quantitative real-time PCR reaction (qPCR).

### Experimental Validation of Identified OBPs and CSPs

The transcripts encoding novel EbalOBPs/CSPs were derived from *E. balteatus* antennae transcriptome data set, which was constructed previously by Prof. Wang Gui-Rong’s group in our laboratory (Wang et al. 2017a). To examine whether these putative OBPs and CSPs were actually expressed in this species, antennal cDNA were used as a template to clone the intact or partial sequences with each specific primer pair (Supp Table 1 [online only]).

DreamTaq DNA polymerase (Thermo Fisher Scientific, Waltham, MA) was used to amplify individual sequences under the following conditions: an initial denaturation step (95°C for 3 min); followed by 38 cycles at 95°C for 1 min, 55°C for 30 s, 72°C for 1 min; and a final extension of 10 min at 72°C. PCR products were gel-purified and subcloned into the pEasy-T3 vector (TransGen, Beijing, China). Then the positive inserts were sequenced at Beijing Genomic Institute (Beijing, China).

### Basic Bioinformatics Analysis

The open reading frames (ORFs) of the identified chemosensory genes were obtained by the ORF Finder Tool at the NCBI (http://www.ncbi.nlm.nih.gov/gorf/gorf.html). Putative signal peptides and their cleavage sites were predicted with the SignalP 4.1 Server (http://www.cbs.dtu.dk/services/SignalP/) (Petersen et al. 2011). The molecular mass (MW) and isoelectric point (pI) of predicted proteins were computed using the Compute pI/MW online program at the ExPASy proteomics server (http://www.expasy.ch/cgi-bin/pi_tool). Multiple sequence alignments were done using DNAMAN 6.0 (Lynnon Biosoft, Canada) with default gap penalty parameters, and then the results were viewed by WebLogo 3.0 for a visualized presentation (Crooks et al. 2004).

### Motif Analysis

The MEME online tool (version 4.12.0, http://meme-suite.org/tools/meme) was used to discover and analyze the OBP and CSP protein motifs (Bailey et al. 2015) based on previous similar reports (Xu et al. 2009, Gu et al. 2015, Zhao et al. 2018) using the parameters minimum width = 6, maximum = 10, and the maximum number of motifs to find = 8. All OBP and CSP sequences used in this study have intact full-length ORFs, and the translated proteins have lengths similar to those of insect OBPs and CSPs. The OBP peptide signal was removed (using PrediSi software; Hiller et al. 2004) before the alignment.

### Tissue Expression Analysis

Expression in different tissues of these 49 OBPs and five CSPs was evaluated by real-time quantitative PCR (RT-qPCR). Primer pairs were designed by Beacon Designer 7.90 (Premier Biosoft International, Palo Alto, CA). The specificity of all primers was confirmed by visualization of a single band amplicon of the expected size after 2% agarose gel electrophoresis and a single peak in a qPCR melting curve; the efficiency was then calculated by analyzing standard curves with a 10-fold cDNA dilution series. In all experiments, all primers gave amplification efficiencies of 90–100%. Primer pairs selected for qPCR analyses and the results of efficiency tests are presented in Supp Table 4 (online only).

The qPCR experiments were subsequently conducted on an ABI 7500 Real-Time PCR System (Applied Biosystems, Carlsbad, CA) using SYBR Green SuperReal PreMix Plus (TianGen, Beijing, China), in a 20 μl reaction volume. The cycling parameters were one cycle of 95°C for 15 min; then 40 cycles of 95°C for 10 s and 62°C for 32 s. For data reproducibility, each reaction was done in three technical replicates on three independent biological replicates. The ribosomal protein S3 gene (*rps3*) exhibits a stable expression across all tissue types, was selected as the reference gene for normalizing target gene expression (Wang et al. 2017a).

The relative transcript level of each target gene among various tissues was calculated using the 2^-ΔΔCT^ method (Livak and Schmittgen 2001), and obtained data were subsequently compared for significant differences (*P <* 0.05) with a one-way nested analysis of variance (ANOVA), followed by Tukey’s honest significance difference (HSD) test, using the SPSS Statistics 18.0 software (SPSS, Chicago, IL).

### Evolutionary Analysis

We analyzed the phylogeny of OBPs and CSPs for two common predatory hoverflies, *E. balteatus* and *E. corollae*, through assessing three principal concepts: nonsynonymous substitutions per nonsynonymous site (Ka), synonymous substitutions per synonymous site (Ks), and the ratio between Ka and Ks (Ka/Ks). KaKs_Calculator software with the MS model was employed to estimate the Ka, Ks values and its ratio Ka/Ks, for obtained putative orthologous pairs between the two species (Zhang et al. 2006). The input files for KaKs_Calculator were prepared by ParaAT (parallel alignment and back-translation) with default settings (Zhang et al. 2012). Orthologous genes in the two closely related species were determined based on our previous phylogenetic analyses (Jia et al. 2019).

## Results and Discussion

### OBP and CSP Genes in *E. balteatus*

In total, 49 assembled transcripts encoding putative OBPs and six encoding CSPs were obtained from previously generated *E. balteatus* antennae transcriptome databases (Wang et al. 2017a). To confirm the validity of these sequences, we first designed specific full-length primers to amplify the ORFs of each gene. As a result, almost all the putative genes, 44 OBPs and six CSPs (Table 1), were successfully amplified and have been deposited in GenBank under the accession numbers MT247210 to MT247259. Though not all OBPs described were cloned from *E. balteatus* tissues, we should point out that these unidentified transcripts were partial sequences (<300 bp).

**Table 1.** Characteristics of OBP and CSP genes identified in *E. balteatus*

Gene	Acc. number	ORF (bp)	SP (aa)	MM (kDa)	pI	Class
EbalOBP1	MT247215	783	1–19	29.54	6.22	Plus-C
EbalOBP2	MT247216	762	1–19	29.96	7.55	Classic
EbalOBP3	MT247217	5’missng	NF	23.89	7.51	Plus-C
EbalOBP4	MT247218	585	1–27	21.75	6.08	Plus-C
EbalOBP5	MT247219	456	1–15	17.72	5.33	Minus-C
EbalOBP6	MT247220	474	1–19	17.76	5.56	Classic
EbalOBP7	MT247221	471	1–15	18.54	4.91	Minus-C
EbalOBP8	MT247222	468	1–16	18.29	5.01	Minus-C
EbalOBP9	MT247223	471	1–15	18.25	5.59	Minus-C
EbalOBP10	MT247224	462	1–19	17.52	4.68	Classic
EbalOBP11	MT247225	456	1–15	17.66	5.33	Minus-C
EbalOBP12	MT247226	453	1–16	17.3	6.09	Classic
EbalOBP13	MT247227	450	1–24	16.57	4.86	Classic
EbalOBP14	MT247228	450	1–17	16.62	5.02	Minus-C
EbalOBP15	MT247229	450	1–15	17.43	6.17	Minus-C
EbalOBP16	MT247230	447	1–15	17.47	5.32	Minus-C
EbalOBP17	MT247231	444	1–24	16.48	8.17	Classic
EbalOBP18	MT247232	441	1–15	17.01	5.91	Minus-C
EbalOBP19	MT247233	441	1–19	16.51	4.58	Classic
EbalOBP20	MT247234	441	1–20	16.99	5.52	Classic
EbalOBP21	MT247235	441	1–16	17.03	5.1	Minus-C
EbalOBP22	MT247236	438	1–23	16.25	8.16	Classic
EbalOBP23	MT247237	435	1–20	16.72	5.44	Classic
EbalOBP24	MT247238	432	1–18	15.42	5.82	Classic
EbalOBP25	MT247239	429	1–18	16.57	9.1	Classic
EbalOBP26	MT247240	429	1–17	16.24	7.56	Classic
EbalOBP27	MT247241	375	1–18	13.87	5.31	Classic
EbalOBP28	MT247242	417	1–19	15.69	5	Classic
EbalOBP29	MT247243	417	1–23	15.79	5.75	Classic
EbalOBP30	MT247244	414	1–20	15.73	5.71	Classic
EbalOBP31	MT247245	5’missng	NF	15.86	5.16	Classic
EbalOBP32	MT247246	408	1–18	14.95	4.45	Classic
EbalOBP33	MT247247	399	1–18	14.41	6.13	Classic
EbalOBP34	MT247248	375	1–18	14.13	5.31	Classic
EbalOBP35	MT247249	396	1–25	14.94	5.94	Classic
EbalOBP36	MT247250	393	1–18	14.79	5.96	Classic
EbalOBP38	MT247251	390	1–18	14.65	6.57	Classic
EbalOBP39	MT247252	390	1–19	14.54	4.69	Classic
EbalOBP40	MT247253	390	1–18	14.34	5.77	Classic
EbalOBP41	MT247254	381	1–18	13.85	5.45	Classic
EbalOBP42	MT247257	—	—	—	—	—
EbalOBP43	MT247258	—	—	—	—	—
EbalOBP45	MT247255	—	—	—	—	—
EbalOBP49	MT247259	—	—	—	—	—
EbalCSP1	MT247256	—	—	—	—	
EbalCSP2	MT247210	384	1–18	14.47	8.81	
EbalCSP3	MT247211	642	1–20	24.42	9.16	
EbalCSP4	MT247212	966	1–20	35.34	8.83	
EbalCSP5	MT247213	339	1–26	12.7	10.05	
EbalCSP6	MT247214	429	1–19	16.18	5.26	

ORF, open reading frame; SP, signal peptide; MM, molecular mass; pI, isoelectric point; NF, not found; dash (—) indicates that gene is partial and has not intact ORF.

Other OBP and CSP physicochemical properties such as molecular mass, isoelectric point, and signal peptide were used to further characterize these identified proteins. Bioinformatic analysis revealed that 38 of the 44 EbalOBPs had intact ORF of 124–260 amino acids long. All identified full-length EbalOBPs had a signal peptide at the N-terminus, ranging from 15 to 27 amino acids. The full-length deduced protein sequences had theoretical molecular masses of 13.85–29.96 kDa and isoelectric points of 4.45–9.1. In the CSP family genes, aside from EbalCSP1, five EbalCSPs contained a complete ORF. EbalCSP5 had the shortest ORF (112 amino acids) with a molecular mass of 12.70 kDa, and EbalCSP4 showed the longest ORF (321 amino acids) with a molecular mass of 35.34 kDa. All the full-length EbalCSPs were predicted to possess signal peptides, varying from 18 to 26 amino acids. These results matched what has been described for other reported insect species (Vieira and Rozas 2011).

**Fig. 1.**
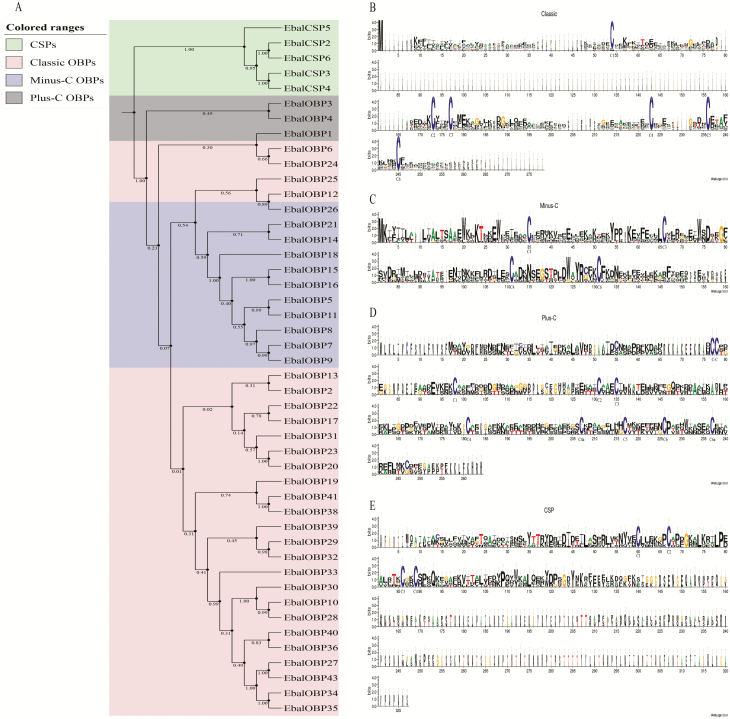
Phylogeny and conserved Cys residues of the OBPs and CSPs identified in *E. balteatus*. (A) Phylogenetic tree constructed using the neighbor-joining method. Bootstrap values were calculated with 1000 replications using MEGA 5.0 (Tamura et al., 2011). (B–E) Sequence logo plots generated from multiple sequence alignment of (B) classic OBPs, (C) minus-C OBP, (D) plus-C OBPs, and (E) CSPs.

The presence of conserved cysteine residues is most characteristic of the OBP and CSP gene families. On the basis of the presence or absence of Cys residues, insect OBPs can be subdivided into distinct classes: classic, plus-C, minus-C, atypical, and dimer OBPs (Hekmat-Scafe et al. 2002, Zhou et al. 2004, 2010; Pelosi et al. 2006; Xu et al. 2009). Here, the full-length sequence alignment showed that among the 38 OBPs, 26 (EbalOBP2, 6, 10, 12, 13, 17, 19, 20, 22–36, 38–41) belonged to the classic class, with the typical six conserved cysteines and fit the motif C1-X22-32-C2-X3-C3-X30-47-C4-X8-10-C5-X8-C6; 10 (EbalOBP5, 7–9, 11, 14–16, 18, 21) falling into the Minus-C category and had four conserved cysteines with C2 and C5 missing, fitting the motif C1-X32-C3-X36-46-C4-X18-C6; the remaining 2 OBPs (EbalOBP1, 4) appeared to be Plus-C OBPs, which have four additional conserved cysteines (a C-C pattern, C4a and C6a) and a conserved proline next to the sixth cysteine, fitting the motif C1-X22-35-C2-X3-C3-X43-C4-X23-25-C4a-X9-C5-X8-C6-P-X9-C6a-X13-26. For the six CSPs, all the predicted amino acid sequences shared the conserved four-cysteine signature and fit the motif ‘C1-X6-C2-X18-C3-X2-C4’. Further phylogenetic analysis confirmed the relationships between all the identified OBP and CSP sequences, showing that the three subgroups of OBPs (Classic Minus-C, Plus-C, and OBPs) and CSPs clearly clustered into distinct clades as hypothesized. The phylogenetic tree and weblogs of alignment of the deduced OBP and CSP protein sequences are shown in Fig. 1.

The validity of 44 *EbalOBPs* and six *EbalCSP*s was verified by cloning and sequencing. Almost all the deduced proteins had the typical characteristics of the insect OBP or CSP families, such as the presence of N-terminal signal peptides and conserved cysteines. Moreover, this species may have larger repertoires of OBPs and CSPs because some studies have proposed that some genes for OBPs and CSPs may be expressed primarily in nonantennae tissues (Sparks et al. 2014, Sun et al. 2017).

### Motif Pattern Analysis of OBPs and CSPs

Conserved motifs are frequently used in analyses of insect OBPs and CSPs due to the importance for functional domains (Xu et al. 2009, Gu et al. 2015, He et al. 2017, Zhao et al. 2018). Hence, we carried out a motif-pattern analysis to compare the motif pattern among OBPs and among CSPs in dipteran insects, using 296 OBPs (from nine dipteran species) and 51 CSPs (from 12 dipteran species), respectively (Supp Table 3 [online only]).

**Fig. 2.**
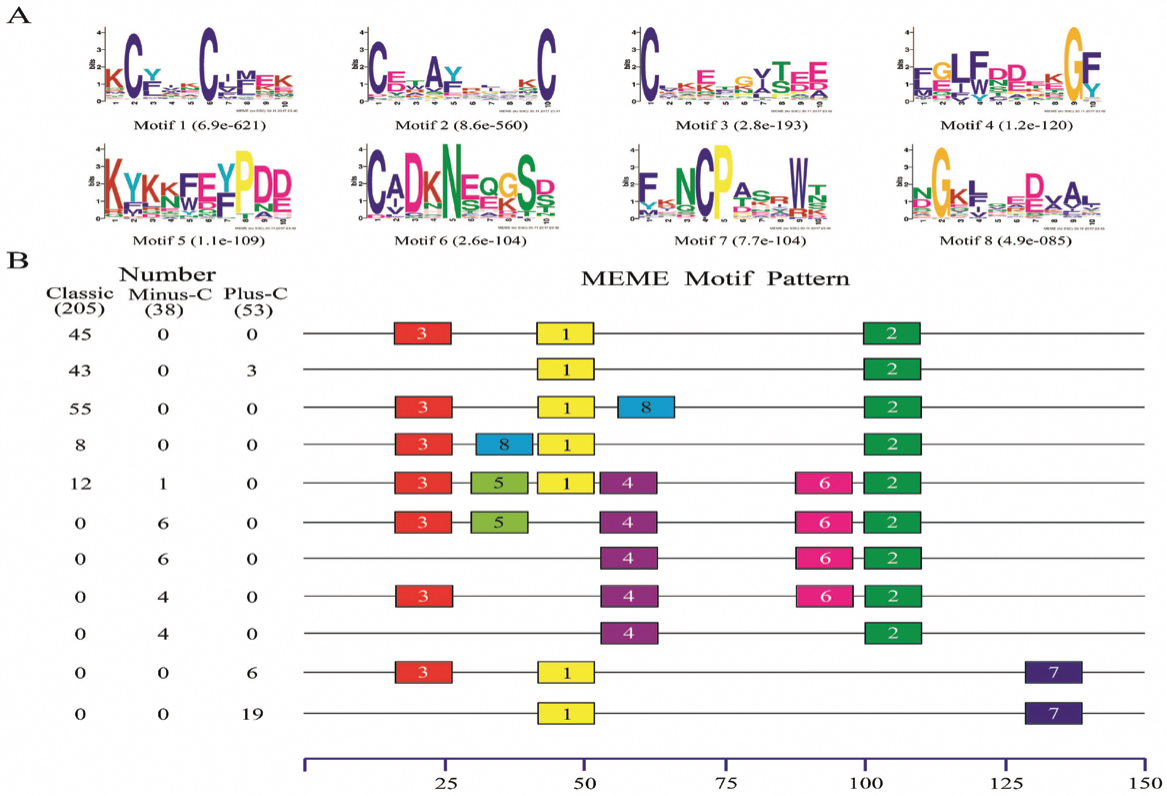
Motif analysis of dipteran OBPs. (A) The eight motifs discovered in the 296 dipteran OBPs by MEME (version 4.12.0; http://meme.nbcr.net/meme/). (B) The numbers in the two columns on the left of the figure are the numbers of OBPs corresponding to the MEME motif patterns on the right. The numbers in boxes correspond to the numbered motifs in (A), where a small number indicates high conservation. The numbers on the bottom show the approximate locations of each motif in the amino acid sequence of the protein, starting from the N-terminal. Only the 10 most common motifs are included, and each motif was present in more than two OBPs. The names of the 296 OBPs, their amino acid sequences, and the associated taxa are listed in Supp Table 2 (online only).

The 68 different motif patterns discovered in the 296 dipteran OBPs, and 11 most common motif patterns present in 212 dipteran OBPs (71.62%) are listed in Fig. 2. As described earlier, three classes of OBPs (classic, minus-C, and plus-C) were found; thus, we also analyzed the motif differences between these classes. The results showed that the motif patterns were quite different among these three classes: motif patterns 3-1-8-2 and 3-1-2 were the most common and detected in 34 and 15 OBPs, respectively. For plus-C OBPs, 53% had three different motif patterns, with the most common being 1–7. In addition, we also found some interesting regular patterns: only the first, second, and third motifs were found in most OBPs. The fourth and sixth motifs were predominately contained in minus-C OBPs, while the seventh appeared in only plus-C OBPs. These structural differences might imply a functional difference between these classes. Thus, despite further functional studies are required, we speculate that these structural differences might be a potential reason the OBPs can bind to ligands of diverse sizes and shapes (Hekmat-Scafe et al. 2002, Pelosi et al. 2006; Zhou et al. 2004, 2010; Xu et al. 2009).

**Fig. 3.**
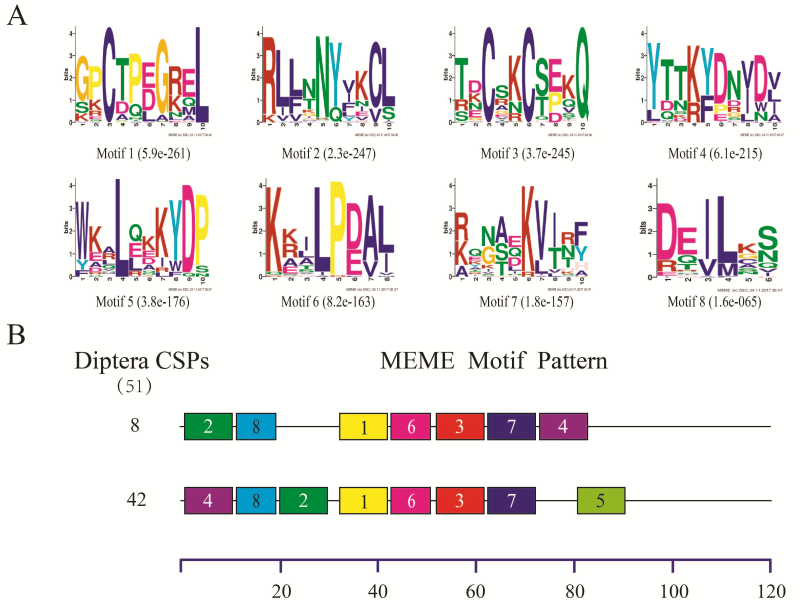
Motif analysis of Diptera CSPs. (A)The eight motifs discovered in the 51 Diptera CSPs by MEME (version 4.12.0) on line server (http://meme.nbcr.net/meme/). (B) Approximate locations of each motif on the protein sequence. The numbers in the boxes correspond to the numbered motifs in the upper part of the figure, where small number indicates high conservation. The numbers on the bottom showed the approximate locations of each motif on the protein sequence, starting from the N-terminal. The protein names and sequences of the 51 CSPs from nine different Diptera species are listed in Supp Table 3 (online only).

Consistent with what has been reported for insects in Diptera (Xu et al. 2009, Zhao et al. 2018) and many other orders (Gu et al. 2015, He et al. 2017), the motif patterns found in CSPs were more conserved than those in OBPs among different insect species. In the 51 dipteran CSPs (from nine species), only three motif patterns were found, and as shown in Fig. 3, 50 CSPs (98.04%) had the two most common motif patterns, with 42 CSPs having motif pattern 4-8-2-1-6-3-7-5, and eight having 2-8-1-6-3-7-4.

### Tissue expression profile of the EbalOBPs and EbalCSPs

**Fig. 4.**
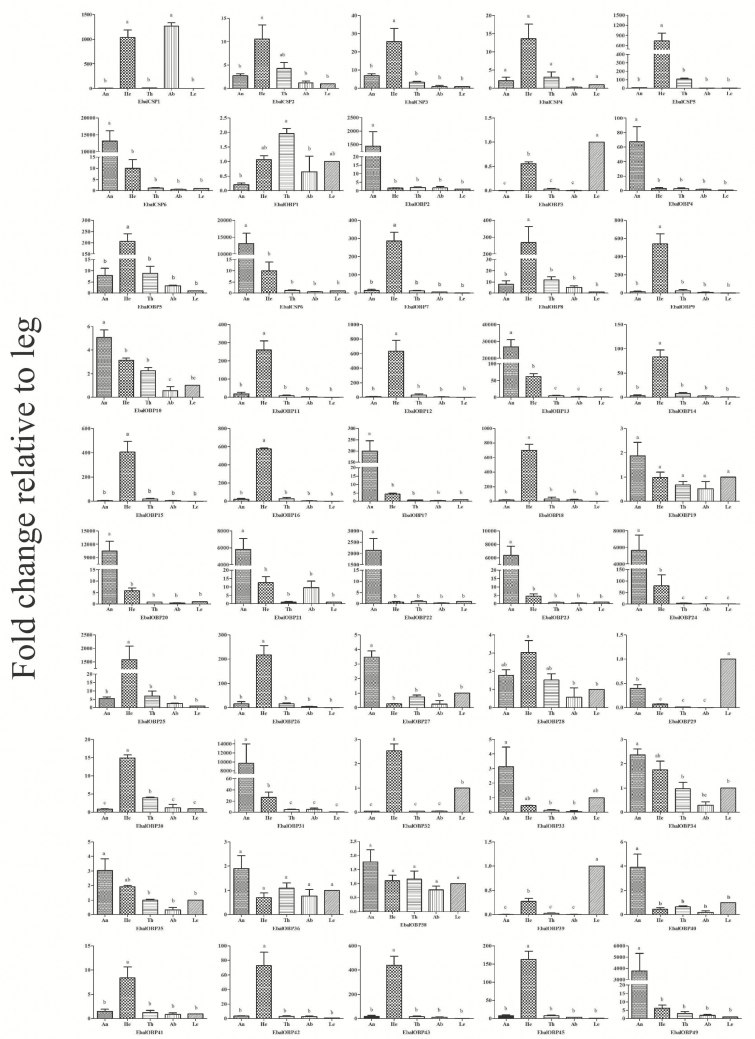
Mean (±SE) transcript levels of OBP and CSP genes in different tissues of *E. balteatus* as evaluated by qPCR. An: antennae; He: head; Th: thorax; Ab: abdomen; Le: leg. Here, the leg was taken as the calibrator or 1× sample. Means are from three biological replicates; the different letters above the bars in a graph indicate a significant difference in expression among tissues (*P* < 0.05).

In the RT-qPCR analyses to measure relative expression of the genes in different tissues (antennae, heads [without antennae], thoraxes, abdomens, and legs) of *E. balteatus* (Fig. 4), most chemosensory genes were broadly distributed in the chemosensory organs such as antennae, heads, and legs, in line with similar analyses (e.g., Cui et al. 2016, Sun et al. 2016, Wang et al. 2017b). Among the 44 EbalOBPs and six EbalCSPs analyzed, 11 OBP genes (*OBP 2, 4, 13, 17, 20, 21, 22, 23, 24, 31,* and *49*) and one CSP gene (*CSP6*) were antennae-specific or enriched, were expressed exclusively in the antennae, with 60- to 10,000-times higher expression in the antennae than in the other body parts; 18 OBPs (*OBP5, 7, 8, 9, 11, 12, 14, 15, 16, 18, 25, 26, 30, 32, 41, 42, 43,* and *45*) and two CSPs (*CSP3, 5*) displayed head-enriched expression; three OBP genes (*OBP3, 29, 39*) had significantly higher expression in the legs than in other tissues; the remaining 12 OBP genes (*OBP1, 6, 10, 19, 27, 28, 33, 34, 35, 36, 38,* and *40*) and three CSP genes (*CSP1, 2,* and *4*) were expressed in more than two tissues, or they showed no significant differences among different tissues.

The antennae-enriched chemosensory proteins are thought to account for odorant recognition and perception (Pelosi et al. 2014, Brito et al. 2016), whereas genes that are highly expressed in gustatory organs, such as mouthparts, legs, proboscis, may be involved in gustatory behaviors. Indeed, such gustatory functions for OBPs in legs have been clearly demonstrated in some insects, such as *Drosophila melanogaster* and *Adelphocoris lineolatus* (Galindo and Smith 2001; Jeong et al. 2013; Sun et al. 2016, 2017). Therefore, the antennae-specific genes obtained here could be potential targets for investigating the molecular mechanisms of chemosensation in adult *E. balteatus*, while the three leg-biased genes might be associated with the detection of nonvolatile substances.

### Evolution Analysis of OBP and CSP Orthologs

An investigation of evolution of chemosensory genes in these closely related species may provide clues about the differentiation and host preference of the predatory syrphids. Positive selection between putatively homologous copies was analyzed in two commonly predatory hoverflies, *E. balteatus* and *E. corollae*. Because OBPs share low sequence similarity, similar to what has been done in other insects (Campanini et al. 2016, Jiang et al. 2017), we performed independent evolutionary analyses on each of the three OBP subfamilies identified (data not shown).

We analyzed three principal concepts that reflect the selection pressure: nonsynonymous substitution rates (Ka), synonymous substitution rates (Ks), and ω rates (Ka/Ks). The estimated ratios of nonsynonymous to synonymous substitutions are listed in Table 2. In accordance with a similar analysis (Campanini et al. 2016, Jiang et al. 2017), all the Ka/Ks ratios were far less than 1.0, indicating that these genes are under strong purifying selection pressure.

**Table 2.** Estimates for Ka, Ks, and Ka/Ks ratio for putatively orthologous OBP and CSP genes from *E. balteatus* and *E. corollae*

Gene pair compared	Ka	Ks	Pairwise Ka/Ks
EbalOBP1–EcorOBP2	0.24416	2.34607	0.10418
EbalOBP1–EcorOBP3	0.49258	3.21105	0.15340
EbalOBP3–EcorOBP4	0.23802	1.83007	0.13006
EbalOBP7–EcorOBP6	0.30803	2.93471	0.10496
EbalOBP8–EcorOBP6	0.30793	1.72943	0.17803
EbalOBP9–EcorOBP6	0.25755	2.17604	0.11836
EbalOBP14–EcorOBP11	0.31790	0.81588	0.38964
EbalOBP17–EcorOBP14	0.00285	0.10202	0.02789
EbalOBP18–EcorOBP7	0.37527	3.53355	0.10620
EbalOBP21–EcorOBP13	0.23399	1.12186	0.20858
EbalOBP23–EcorOBP15	0.02784	0.25389	0.10966
EbalOBP24–EcorOBP17	0.20356	1.60117	0.12713
EbalOBP27–EcorOBP23	0.00329	0.06645	0.04947
EbalOBP31–EcorOBP20	0.05299	1.24151	0.04268
EbalOBP32–EcorOBP25	0.23657	1.24667	0.18976
EbalOBP33–EcorOBP27	0.00004	0.03924	0.00000
EbalOBP34–EcorOBP23	0.18258	0.79731	0.22900
EbalOBP35–EcorOBP23	0.17179	0.91638	0.18746
EbalOBP36–EcorOBP32	0.00313	0.01555	0.20153
EbalOBP40–EcorOBP33	0.01126	0.02771	0.40659
Median	0.21878	1.18169	0.12859
EbalCSP2–EcorCSP1	0.02895	0.56276	0.05144
EcorCSP5–EbalCSP3	0.09714	0.73580	0.13201
EbalCSP3–EcorCSP6	0.17875	1.10239	0.16215
EbalCSP3–EcorCSP7	0.20553	1.12375	0.18292
EbalCSP4–EcorCSP5	0.20166	1.16534	0.17305
EbalCSP4–EcorCSP6	0.28812	1.52324	0.18915
EbalCSP4–EcorCSP7	0.13822	0.90006	0.15357
EbalCSP5–EcorCSP4	0.01257	0.56174	0.02237
EbalCSP6–EcorCSP2	0.06799	0.70548	0.09637
Median	0.13822	0.90006	0.15357

### Conclusions

In this work, 44 OBPs and six CSPs from *E. balteatus*, based on previously reported antennal transcriptomic data, were directly cloned, and sequence alignment, phylogenetic analysis, and conserved motif identification indicated that all identified genes have typical characteristics of the insect OBP or CSP family. More importantly, some antennae-specific genes that may be involved in chemical cue recognition were identified through RT–qPCR. The next step, therefore, is functional analysis of these candidate OBPs/CSPs through electrophysiological studies and gene expression modification studies to reveal their roles in chemoreception and further explore the molecular mechanisms chemoreception in *E. balteatus*. Such studies should contribute to understanding the tritrophic interactions among plants, the herbivorous insects that feed on the plants, and the natural enemies of the pests.

## Supplementary Material

ieaa065_suppl_Supplementary_Material
