# Supplementary material for: Odorant Binding Proteins and Chemosensory Proteins in Episyrphus balteatus (Diptera: Syrphidae): Molecular Cloning, Expression Profiling, and Gene Evolution
Source: J Insect Sci. 2020 Aug 8;20(4):15. doi: 10.1093/jisesa/ieaa065 (PMC7414794; doi:10.1093/jisesa/ieaa065)
Supplement: ieaa065_suppl_Supplementary_Material [file ieaa065_suppl_supplementary_material.docx]

**Odorant binding proteins and chemosensory proteins in *Episyrphus balteatus* (Diptera: Syrphidae): molecular cloning, expression profiling and gene evolution**

Hui-Ru Jia^1#^, Lin-Lin Niu^1#^, Yu-Feng Sun^2^, Yong-Qiang Liu^1^, Kong-Ming Wu^1^*

1. State Key Laboratory for Biology of Plant Diseases and Insect Pests, Institute of Plant Protection, Chinese Academy of Agricultural Sciences, Beijing, 100193, China
2. Laboratory of Agro-products Quality Safety Risk Assessment (Beijing), Institute of Food Science and Technology, Chinese Academy of Agricultural Sciences, Beijing 100093, China

(*) Correspondence:

Kong-Ming Wu, State Key Laboratory for Biology of Plant Diseases and Insect Pests, Institute of Plant Protection, Chinese Academy of Agricultural Sciences, Yuanmingyuan West Road, Haidian District, Beijing 100193, China. E-mail: kmwu@ippcaas.cn; Tel.:+86 10 8210 5551; Fax: +86 10 8210 5551.

# These authors contributed equally to this work.

**Supporting Information**

**Table S1**.

Primers used for molecular cloning of *E.balteatus* OBP and CSP genes.

**Table S2**.

Protein names and sequences of the 296 OBPs used in Figure 2.

**Table S3**

Protein names and sequences of the 51 CSPs used in Figure 3.

**Table S4**.

Primers used in RT–qPCR for quantifying expression levels of OBP and CSP genes from *E. balteatus*.

**Table S1**.Primers used for molecular cloning of *E.balteatus* OBP and CSP genes.

| Gene | Forward primers (5'-3') | Reverse primers (5'-3') |
| --- | --- | --- |
| CSP1 | ATGGTTCTAGCCAACAAAC | TTACGCAAATGTTCGACAA |
| CSP2 | ATGAAGGTACTCATCGTA | TTAAGCCTTAACTCCAAG |
| CSP3 | ATGCAGGCGATAGCAATT | AATGGAATATTTGCCAAAATGC |
| CSP4 | GTGTAGCGTGTTGTTTGT | TTAACTCCGCTGTGGCTG |
| CSP5 | ATGTCCATTAAGATGCCGTC | AAACACCTCGATATTTTCTTATC |
| CSP6 | ATGTTCGGTCGTACAATT | TTATTTCTCTGATTCCTTTGTC |
| OBP1 | TACCCAAGTTATAATACCTT | CAATATTTTCAATGACGATG |
| OBP2 | ATGATAGCATTTCAAAGC | TCATGTAAAAATCAACGTAT |
| OBP3 | ATGTCAAACTATCTCAATCTAC | TTATTTTGGTGGTGGAGG |
| OBP4 | ATGGATCACTACGGAAGA | AAAACAGAACACTTTTGTAAA |
| OBP5 | ACATTGCACTTAAACTTC | TTTATCACACAAAATCCC |
| OBP6 | ATGAAGAAATATACAATTG | TCAAATTTGTTTTATATCG |
| OBP7 | CCGTTTCACAAATACTTT | TTCTTAGACTATTCCTTCG |
| OBP8 | ATGAAAATTTATATAATTTTAACAT | TCACCATTCATAATTCAG |
| OBP9 | ATGAAAATTTATATAATTTTAGCA | CTATTCCTTTGAATCAACA |
| OBP10 | AATTAGTTAGAAATGAAGTTC | ATAATAATGGTAGTGATGTC |
| OBP11 | ATGAAAATTTGTATTATTTTAGCA | TTATGCATTTACTGTTTCC |
| OBP12 | ATGAAATTCTTTATTGCAATTGTA | TTAATGATGATGGGCAGA |
| OBP13 | ATGTTGTCGGTTAGATTT | CTAAATGACTTGTTTGTCT |
| OBP14 | TTAATCAACCTGAACAGA | GCATTATTATCCACACTTAT |
| OBP15 | ATGAAATTATTTATCATTTTGGC | TTAGTTTTCCGGTTCTTC |
| OBP16 | ATGAAAATCTATATCATTTTAGCA | TTAGTGTTCCTTGTGATC |
| OBP17 | ATGTTTAAGGATTCTACAATCTG | TTAAGGGAATTTAAAACTCGG |
| OBP18 | ATGAAATCGTTTATAATTTTAAC | TCATTCCTGGTAAAAAGA |
| OBP19 | ATGAAGAATATAGTTTCCTTA | TTATTGTTTACTGTCTTCC |
| OBP20 | TAGCCTATCTCTACATTG | AGTGAGTCTCTTCATATC |
| OBP21 | ATGAAAACTTTCCTAGTTG | ATTTGGTACTCCATGAAT |
| OBP22 | ATGTCGTTTCTTCTAAAAC | TTAAGGAAACATAAATCTAGG |
| OBP23 | ATGAAAATATTTATTCCGTTT | TTAAGGTAGAAAATAATGCA |
| OBP24 | ATGAAATTAATAAGTTTCATAG | TTAAATCAAACCATTGACTC |
| OBP25 | AATCAGCAAATATGAAGTA | AATTCTTTCCTACACATTT |
| Obp26 | ATGAGATTTACTATTGCA | TTATGCTACATTCTTCTT |
| OBP27 | ATGAAATTCTTAATACTTGTAG | TCAAACCTTCTTCTGTAA |
| OBP28 | ATGAAGTATCTTATTTTTATTAGT | TTATTCACTCAACGAAAA |
| OBP29 | ATGAAGTCAGTCATTGCA | TCAATACTTGAAAATATCACTC |
| OBP30 | ATGAAGTTCTTCATAATATTTATTG | TTATGAACTATAAATTTGAGTAAT |
| OBP31 | ATGGACAAATATATGATTT | TTAAATTAAAATCGCATTC |
| OBP32 | ATGAAGTTTGTTCTGATC | CTAAATTTTGAAGATATCATCA |
| OBP33 | ATGAAATTCTTTTTGGCTAT | TTAATAGGCATTATTTTTCTTG |
| OBP34 | ATGAAATTCTTAATTATTGTTGTA | TCAAACTTTCCTGCTTAA |
| OBP35 | ATGAAATTGAATTTATTAAAAATGA | TCAAACTTTCCTGCTTAA |
| OBP36 | ATGAAATTATTAATAGTTCTTGC | CTAGTAGGCTTTATTTTTGT |
| OBP38 | ATGCAATATTTCATTATTGTAAT | TTACATATATTTTCTTAAATCATTT |
| OBP39 | CATTAAAAGTTTAAAATATGAAGT | GAATGAGAACTAATTGAAGA |
| Obp40 | ATGAAATTCTTCATTATTATTG | CTAATAGGCTTTATTTTTGA |
| OBP41 | ATGAAGTTCTTTATTGTTG | TTATTTGTTATGGAATTTGG |
| OBP42 | ATGAAGTTCATCATTTTAGTTAC | TTTGACGAAACATTGGTG |
| OBP43 | GTTATCAACATGACTAGCA | CTTGAACAACTCATACCC |
| OBP45 | ATGCTCGCCGCCTTGGCATCAG | TTAATCTAGCTTATCAAAGC |
| OBP49 | TTACTTAAGTGAAACCAAA | ATTCCTTTTCCAATGTTG |

**Table S2**. Protein names and sequences of the 296 OBPs used in Figure 2.

>DmelOBP19d

MSHLVHLTVLLLVGILCLGATSAKPHEEINRDHAAELANECKAETGATDEDVEQLMSHDLPERHEAKCLRACVMKKLQIMDESGKLNKEHAIELVKVMSKHDAEKEDAPAEVVAKCEAIETPEDHCDAAFAYEECIYEQMKEHGLELEEH

>DmelOBP19a

MGLESGIRQIVSEMKFHLLLVCVAISLGPIPQSEAGVTEEQMWSAGKLMRDVCLPKYPKVSVEVADNIRNGDIPNSKDTNCYINCILEMMQAIKKGKFQLESTLKQMDIMLPDSYKDEYRKGINLCKDSTVGLKNAPNCDPAHALLSCLKNNIKVFVFP

>DmelOBP76a

MKHWKRRSSAVFAIVLQVLVLLLPDPAVAMTMEQFLTSLDMIRSGCAPKFKLKTEDLDRLRVGDFNFPPSQDLMCYTKCVSLMAGTVNKKGEFNAPKALAQLPHLVPPEMMEMSRKSVEACRDTHKQFKESCERVYQTAKCFSENADGQFMWP

>DmelOBP57a

MFNTRLAIFLLLIVVSLSQAKESQPFDFFEGTYDDFIDCLRINNITIEEYEKFDDTDNLDNVLKENVELKHKCNIKCQLEREPTKWLNARGEVDLKSMKATSETAVSISKCMEKAPQETCAYVYKLVICAFKSGHSVIKFDSYEQIQEETAGLIAEQQADLFDYDTIDL

>DmelOBP56f

MKVFLLFIFISAIWLQAFCMKSSEKIKACLKRQLGYTITENTKFDAKEDSLQSKCFYHCLLEVKGVIANDAISSEQPRKVLEKKYGITDTDELEKAEEKCHSIKASGKCELGYEILKCYQSITKH

>DmelOBP56b

MKLIYLLVVFLIFALSELVAGQSAAELAAYKQIQQACIKELNIAASDANLLTTDKEVANPSESVKCYHSCVYKKLGLLGDDGKPNTDKIVKLAQIRFSSLPVDKLKSLLTSCGTTKSAATCDFVYNYEKCVVKGISA

>DmelOBP83b

MVKYPLILLLIGCAAAQEPRRDGEWPPPAILKLGKHFHDICAPKTGVTDEAIKEFSDGQIHEDEALKCYMNCLFHEFEVVDDNGDVHMEKVLNAIPGEKLRNIMMEASKGCIHPEGDTLCHKAWWFHQCWKKADPVHYFLV

>DmelOBP22a

MRVLLAFVLLLGLSVLATKEPEEVKIVSECAKENNVHRKKALDLLMSYRLKKKTHNVMCFINCIFERTNILQKVKEKVVKENHNCDSIKDADKCAESFQKFQCLVKIEMKVRGIDRG

>DmelOBP69a

MVARHFSFFLALLILYDLIPSNQGVEINPTIIKQVRKLRMRCLNQTGASVDVIDKSVKNRILPTDPEIKCFLYCMFDMFGLIDSQNIMHLEALLEVLPEEIHKTINGLVSSCGTQKGKDGCDTAYETVKCYIAVNGKFIWEEIIVLLG

>DmelOBP57e

MLDQLTLCLLLNFLCANVLANTSVFNPCVSQNELSEYEAHQVMENWPVPPIDRAYKCFLTCVLLDLGLIDERGNVQIDKYMKSGVVDWQWVAIELVTCRIEFSDERDLCELSYGIFNCFKDVKLAAEKYVSISNAK

>DmelOBP56e

MKVFFVFAALAALSLASAVGLTDSQKAEAKQRAKACVKQEGITKEQAIALRSGNFADSDPKVKCFANCFLEQTGLVANGQIKPDVVLAKLGPIAGEANVKEVQAKCDSTKGADKCDTSYLLYKCYYENHAQF

>DmelOBP83a

MALNGFGRRVSASVLLIALSLLSGALILPPAAAQRDENYPPPGILKMAKPFHDACVEKTGVTEAAIKEFSDGEIHEDEKLKCYMNCFFHEIEVVDDNGDVHLEKLFATVPLSMRDKLMEMSKGCVHPEGDTLCHKAWWFHQCWKKADPKHYFLP

>DmelOBP84a

MYSALVRACAVIAFLILSPNCARALQDHAKDNGDIFIINYDSFDGDVDDISTTTSAPREADYVDFDEVNRNCNASFITSMTNVLQFNNTGDLPDDKDKVTSMCYFHCFFEKSGLMTDYKLNTDLVRKYVWPATGDSVEACEAEGKDETNACMRGYAIVKCVFTRALTDARNKPTV

>DmelOBP19b

MMQCSRMTTTLKMTNLLLAVACAAVLMGSATADEEEGSMTVDEVVELIEPFGDACTPKPSRENIVEMVLNKEDAKHETKCFRHCMLEQFELMPEDQLQYNEDKTVDMINMMFPDREDDGRRIVKTCNEELKAEQDKCEAAHGIAMCMLREMRSSGFKIPEIKE

>DmelOBP47a

MNRVLVLLLVLKMFALSESRFAKININLGLTVADESPKTITEEMIRLCGDQTDISLRELNKLQREDFSDPSESVQCFTHCLYEQMGLMHDGVFVERDLFGLLSDVSNTDYWPERQCHAIRGNNKCETAYRIHQCQQQLKQQQQNLLATKEVEVTTTPAGSDETKP

>DmelOBP57d

MPEKMSLRLVPHLACIIFILEIQFRIADSNDPCPHNQGIDEDIAESILGDWPANVDLTSVKRSHKCYVTCILQYYNIVTASGEIFLDKYYDTGVIDELAVAPKINRCRYEFRMETDYCSRIFAIFNCLRQEILTKS

>DmelOBP56g

MRATFALTLLLGCLSGILAQQANIDSSVSKELVTDCLKENGVTPQDLADLQSGKVKAEDAKDNVKCSSQCILVKSGFMDSTGKLLTDKIKSYYANSNFKDVIEKDLDRCSAVKGANACDTAFKILSCFQAAN

>DmelOBP18a

MKVVCSIAVLWICLITMWQSAGRVNAEGCLKHHNLTSAQVQAVAPSTPVADVPVAVKCYSRCLIQDYFGDDGKIDLQKVGKRGSQEDHVILSQCKQQFDGVTNLDTCDYPYLILQCYFKGKQSGTIAS

>DmelOBP57c

MLKLWLICILTVSVVSIQSLSLLEETNYVSDCLASNNISQAEFQELIDRNSSEEDDLENTDRRYKCFIHCLAEKGNLLDTNGYLDVDKIDQIEPVSDELREILYDCKKIYDEEEDHCEYAFKMVTCLTESFEQSDEVTEAGKNTNKLNE

>DmelOBP56h

MKFTLFCIALAAFLSMGQCNPDFRQIMQQCMETNQVTEADLKEFMASGMQSSAKENLKCYTKCLMEKQGHLTNGQFNAQAMLDTLKNVPQIKDKMDEISSGVNACKDIKGTNDCDTAFKVTMCLKEHKAIPGHH

>DmelOBP19c

MKPSTPVAAIPLMTIVVAVLLQTHCVRGQTQAFDLAKLLPKTGTEPIWAVIDRNLPQVQELVTAARMECIQKLQLPRDQRPLGKVTNPSEKEKCLVECVLKKIKLMDADNKLNVGQVEKLTSLVTQDNKMAIAVSSSMAQACSRGISSKNPCEVAHLFNQCISRQLERNNVKLVW

>DmelOBP56a

MNSYFVIALSALFVTLAVGSSLNLSDEQKDLAKQHREQCAEEVKLTEEEKAKVNAKDFNNPTENIKCFANCFFEKVGTLKDGELQESVVLEKLGALIGEEKTKAALEKCRTIKGENKCDTASKLYDCFESFKPAPEAKA

>DmelOBP56d

MKFLIVLSVILAISAAELQLSDEQKAVAHANGALCAQQEGITKDQAIALRNGNFDDSDPKVKCFANCFLEKIGFLINGEVQPDVVLAKLGPLAGEDAVKAVQAKCDATKGADKCDTAYQLFECYYKNRAHI

>DmelOBP57b

MFIYRLVFIAPLILLLFSLAKARHPFDIFHWNWQDFQECLQVNNITIGEYEKYARHETLDYLLNEKVDLRYKCNIKCQLERDSTKWLNAQGRMDLDLMNTTDKASKSITKCMEKAPEELCAYSFRLVMCAFKAGHPVIDSE

>DmelOBP28a

MQSTPIILVAIVLLGAALVRAFDEKEALAKLMESAESCMPEVGATDADLQEMVKKQPASTYAGKCLRACVMKNIGILDANGKLDTEAGHEKAKQYTGNDPAKLKIALEIGDTCAAITVPDDHCEAAEAYGTCFRGEAKKHGLL

>DmelOBP51a

MKVFIGLVLLLAVTTLSSALFESEANECAKKLGITPDYFENFPHSSRVKCFYHCQMEKLEIIANGVVTPFDLKVLNISPESYDKYGVKVKPCLKLSHRDKCELGYLVFQCLKREFNL

>DmelOBP56i

MHFFTCCALLLVVVTLPTCFVQAGPIKDQCMAAAGITAQDVANRHETDDPGHSVKCFFRCFLENIGIIADNQIIPGAFDRVLGHIVTAEAVERMEATCNMIKSETSHDESCEFAWQISECYEGVRLSDVKKGQRTRNHRG

>AgamOBP1

MKLVTFVFAALLCCSMTLGDTTPRRDAEYPPPELLEALKPLHDICLGKTGVTEEAIKKFSDEEIHEDEKLKCYMNCLFHEAKVVDDNGDVHLEKLHDSLPSSMHDIAMHMGKRCLYPEGETLCDKAFWLHKCWKQSDPKHYFLV

>AgamOBP2

MLAQASPLLLLLLLLVTQCLDGANCSTITTQRPAPRRDGQYPPPETLAFLRPLGKLCLEETGVSPEAIKRFSDADPFDDNRALKCYMDCMFRVTNVTDDRGELHMGKLLEHVPTEFEDIALRMGVRCTRPKGKDVCERAFWFHKCWKTSDPVHYYLV

>AgamOBP8

MPSRKRLCRLLLLLLLPVDLELISQDATHGAYVVRTFADATAYRDECVQQYAGRGSSLIDYMRQVALHTDNADSRWCIVRCILQKADLLDGEGAPHEANVHAQMQHSNAIVEDPDDIRSETSRCLREPPAPDSGGGCLRAYTFFACIQSTEYDLF

>AgamOBP9

MLKFVVALLAFTAVVSAEFVVQTREDLLAYRAECVKSLGVSDELVEKYKSWNFPEDDTTQCYIKCIFNKMQLFDDTNGPIVDNLVVQLAHGRDANEVREEIVKCAGSNTDGNVCHWAFRGFQCFQKNNLSLIKASVKKD

>AgamOBP11

MIKPFVCILIVAAGCANAFMYKHPYNHHQAAVLAHEPVVPVEFVKHTTSPAFRPASFLEVMEVVLDCFNTLRIPLQRFPSYLSGIFPEDPETKCFLRCVAIKLGVYCDEKGADLDRHCVQFGLGECCENFSNRHLVCLQQNSLPCPDRCTAAYKQELCFQEPIAKYLDYHFHDLVGLLHQAKCSHDLKMLHP

>AgamOBP12

MAPVRYHFVLWLLILIGVSSLVPPGECLDISKVTLDAAFYPLFGCARDLVVPEDLIELYKKRIFPDDQLTCCVFRCLGMRLGIYDDVKGFDVDKQYERVKDRLSVDEDTYKRGVKNCIRNVLRGRTLNNCEKAYLILNQCQGNTITNSLNQQLNEIRCN

>AgamOBP15

MLTIVVATSICLMATASANAPKSLSPELLQQMGQFRSECLRETGTTDEQIEQFNSPQSVQASHELQCYMYCMFRLHNVTRPNGELDLIDVYHAIPKQFNSIALKVLAKCNKSTGPIADACERAYSHHRCWKETEPELRLPVAVCLMF

>AgamOBP17

MKLVTFVFAVLVCCSMTLGDTTPRRDAEYPPPELLEALKPLHDICLGKTGVTEEAIKKFSDEEIHEDEKLKCYMNCLFHEAKVVDDNGDVHLEKLHDSLPSSMHDIAMHMGKRCLYPEGETLCDKAFWLHKCWKQSDPKHYFLV

>AgamOBP18

MTSTSNTVTWVVVAVGVYCLVFQPALVNAQQSLTQADMDEIAKGMRKVCMSRHKISEEMANYPSQGIFPDDKEFKCYVACLMDLTQTSKKGKLNYDAAVKQIEILPENYRQPFRLGLDSCRTAADDATDRCEVAYILLKCFFKASPKFFFP

>AgamOBP19

MIISDDPIALWLPILLSITQEQLEKTARTFRQVCQPKHKISDEVADAVNRGVFADTKDFKCYVSCLLDIMQVARKGKVNYEKSLKQIDTMLPDHMKPAFRAGLEACKSAAQGVKDHCEAATILLQCFYKNNPKFVFP

>AgamOBP20

MSWKARNVKRIFPLRKCLWIGSLLLLSTAPGLPPKATVEQMMKSGEMIRSVCLGKTKVAEELVNGLRESKFADVKELKCYVNCVMEMMQTMKKGKLNYDASVKQIDTIMPDELAGPMRAALDICRTVADGIKNNCDAAYVLLQCLSKNNPKFIFP

>AgamOBP21

MQSLQIVFVVLLAAVSTMEQHEIAKSLAEQCRAELGGELPEDFATKMRLGDLTLDSETAKCTIQCMFAKVGFTLESGAANRDVLIAKLSKGNPTAKAEAFADVCENNEGETACDKAFSLYQCYHKNKSIFD

>AgamOBP22

MNSLLLIGGVLVVLNVQFVTAADNNESVIESCSNAVQGAANDELKVHYRANEFPDDPVTHCFVRCIGLELNLYDDKYGVDLQANWENLGNSDDADEEFVAKHRACLEAKNLETIEDLCERAYSAFQCLREDYEMYQNNNNATSE

>AgamOBP23

MKSFFCVASFFLLVASVHAFTLRQQKMVSIFALECMAETGIGAESLTKLRDGDLTANDRTAKCFMKCFFEKENFMDAEGKLQLEAIATALEKDYERAKIDEMLEKCGEQKEDACETAFNAYACYHDHYQNL

>AgamOBP24

MKCSETIVTFAVVLTLLACTVAGAKGARLEAEHVRRIHQNARECVKETGILPKNAFRVLSGDFSVDTMKAKCFVKCFLDKAGFIDDDGVIQQDVIREKLTVGIEAGKVNELIKKCSVEGTDACDTAYQMYKCFFSNHKVPKELFQMRKGIGRRNMQQ

>AgamOBP26

MKTFVAIAVVALIAGTFALTIDQKKKAEGYAAECVKTTGVPPETAAKLKGGDFAGADDKTKCFAKCFLEKAGFMTDKGEIDEKTVIEKLSVDHDRAKVEGLVKKCNHKEANPCETAFKAYQCIYAAKGAVV

>AgamOBP28

MKLLFATVLLAVCAAAQPLTDDQMKKAEGFALGCLEQHKGLNKEHLVLLRDGDFSKVDADTKCFLRCFLQQANFMDAAGKLQNDYVIERLSLNREKSKVEALVKKCSAGVEVEDSCETAFRAVECYHREKASLL

>AgamOBP58

MKTIACLVLASAFIACAVATISEEQREAARQLAGKCMQQTGASEDDVNRLRSGDTEGADRNTRCFVQCFFQGAGFVDQDGSVQTDELTQKLASEYGQEKADELVARCRNNDGPDACERSFRLLQCYMENRASLMF

>AgamOBP59

MGAFESGLGLLGWVAFGMVLLLAGRGCHAQDFKGAIDHCTKDFEMDMDIVVSLKYGDFTERDPLIECFTECLMKKSGFMYDDYTYNKTLIIGFAGRYLEPEGAQAVYDNCIDRFGQTVCVTGFEMYQCIHETAVSEWVSSNF

>AgamOBP81

MVPHQRTDSRWRLILLLLLSFNCCEALPFQVEGQPVVHCNQPPLEVHPKDCCQLPSLIDEELLRNCKTLYGGEPLQRKLIYERGKCFVECALNATGTLVGGVLDQAKILHVIVTATQNDPAVMQLFQSSTLQCFQTVGAGGGGASSPAGCSSLGVDFVGCVNIKNFVNCPPHIWSNSAQCNALRQYILECPQPF

>AgamOBP82

MDENTPQKRCVSRAVTVGICGAIVLLLLVGTSPAPVEGLRCRTGEGPSADDVKRIVRTCMNKITNAGSGGNSSSSSSSTIERDRACLMQCFFEEMKATNADGFPEKHKVLHVITKDIREHELREFYVDSIQECFHMLGLDNRLKDKCDYSMRFVTCLSDRFETNCDDWESVTSAMF

>AgamOBP27

MGRLDLVCLLAIVLLVHSCNGQDILGSYFRCRNEFEIEPSVFESLRAGNFSVRNSLVECFGECFVKRAGFMNDNFTFNRDTIMRFTNRFVSKEISEKVYNICTDNVTPTYCVTAFDVYQCIYENVYKSWDSRK

>AgamOBP4

MSVSVLVSSLVVLFCVQCLIEHIDGAMTMKQLTNSMDMMRQACAPKFKVEEAELHGLRKSIFPANPDKELKCYAMCIAQMAGTMTKKGEISFSKTMAQIEAMLPPEMKTMAKEALTHCKDTQTSYKDPCDKAYFSAKCAADFTPDTFMFP

>AgamOBP6

MTSNAFYSSNTVTWVVAVIGVYCLVFRPALVHAQQSLTQADMDEIAKGMRKVCMSRHKISEEMANYPSQGIFPDDQEFKCYVACLMDLTQTSKKGKLNYDAAVKQIDILPENYRQPFRLGLDSCRTAADDATDRCEVAYILLKCFFKASPKFFFP

>AgamOBP7

MCEYSNTRNKMSNLVVVLVLLTMYIVLSAPFEIPDRYKKPAKMLHEICIAESGASEEQLRTCLDGTVPTAPAAKCYIHCLFDKIDVVDEATGRILLDRLLYIIPDDVKAAVDHLTRECSHIVTPDKCETAYETVKCYFNAHDEVIKFCHLLVLE

>AfraOBP57c

MYQFGVDRENATERGEMYSSGKGMALPRLTWLVIFAAAILFLMPLGRVTATPTGAPSSFVEACLEQHKITEEELDELPDDPNPEDIDMKYKCYANCLLNGLGFMDENGKLNAEMMHEAGILNDDSYEDMIECKAANDMEDDPCEYSFGVMLCARMIHANDEYDEYAENVPMVREVIM

>AfraOBP56h-2

MNSFITVALIVVFSATTLCQPHDPEMRKIVEECNKEHNVSPKDFHEFMEGKLATPSNDLKCSMQCAMVKQGIMTESGTFNADAAKAKMPSDAKLASAIDACKNEAGSSPCDTAAKITQCLVAHK

>AfraOBP56h-1

MRTLYLFTVLAAFVTVLMCQSQADMEKLHKLCMNETNLTEAEIRQFFGNGMKASDAKDNMKCHTKCLMEKQGIIKDGVYVPSVAIKQLMLFPALKGHETEVTQAVNNCKYEKGANECDTAFKITMCIKEFKSHAP

>AfraOBP56d-2

MKVFVIAAVLAFVAAAVAQEGIKLNEAQHKRVRGIANDCIKQTGTTEAAVLNLRAGGFSAVDENAKCFAKCFQEHLGYVKNGSVDEAAVNKSLGPLAGEEKVKAVQAKCNGATGSNDCDAALERYKCYYGENVKNLS

>AfraOBP19d

MTAFSCFLIFITMTSLPTILLADLQPPHYGPLRAMTEAAIEDCYEDAAQKVKVEITDEGFEELLKGSRDNLMHNTKCLRYCIMRKNGLFNASNSLDKEKLVDIFEIIHPQVEKEKLLNVLQKCAEKTEKETDNCERASVAAMCVLDELKGEGVTNI

>AoblOBP19a

MSRKLIFCILITVFIILVLQVTRISGGATEEQMIAAGKLMRDVCLPKFSKVSTEVADGIKLGNVPDTKDVKCYINCVMEMMQTMKKGKFLLESSLKQVDLLMPDDYKDEYRNGLTNCKDAANGIKNNCDASYALLICMRDQIKKFMFP

>AoblOBP56a

MKSTISCCILATVLLCLCTTNTDAFGRKPKKLNPELEAKFDVLTAWIAYRLNLKHAKEACVGEYGFTDELATNLVKIKVANPNDREKCYVNCLYTKLIFYKNNSINTQAMKESLSEIVGEERLLNIVNSCLNVGGANDCEKVYKFHACASPEFDKVREDIFQPDD

>AoblOBP19b

MKMLQTVRIFTGTILLIFTGDVLADEMMNLPMGLLIEAVEPYAINCDPKPELEHAQELFLNKEDAQHTTKCLRRCLMDQFELFIDGGTQVNSGKLVGYMLLAYPDKMEELNEISNGCNEQNEEMGIDEKCEVAHVFGMCMLKEMQSREYEIPKVVQ

>AoblOBP57c

MYQFGMDRENATERGEMYSSGKGMALPRLTWLVIFAAAILFLMPLGRVTATPTGAPSSFVQACLEQHKITEEELDELPDDPNPEDIDMKYKCYANCLLNGLGFMDENGKLNAEMMHDAGIINDDSYEDMIECKAANDMEDDPCEYSFGVMLCARMIHTNDENDEYAEAVPMAREVIM

>AoblOBP56h-2

MNSFITVALLLVFSATTLCQPHDPEMRKIIEECNKEHNVSPKDFHEFMEGKLATPSDDLKCSMQCAMVKQGIMNDSGTFNADAAKAKMPNDAKLASAVDACKNEAGSSPCDTAAKITQCLVAHK

>AoblOBP56d-2

MKVFVIAIAFAFVAAAVAQDSLGLSEEQIQKVHALAGECIKETGTNPAAVRKLRAGDFSEVDENVKCFAKCFQERLGYLKNGSVDEDAVNKSLGPLAGEEKVKAAQAKCNSVTGSNDCDAALERYKCYYGENVKNLS

>AoblOBP56h-1

MRTLYLFTVLAAFVTVLMCQLQADMEKLHKLCMNETNLTEAEIKQFFGNGMKASDAKDNMKCHTKCLMEKQGIIKDGVYMPSVAIKQLMLFPALKGHETEVTQAVNNCKNEKGANTCDTAFKITMCIKEFKSHAP

>AoblOBP56d-1

MKVFVIAIAFAFVAAAVAQDSLGLSEEQIQKVHALAGECIKETGTNPAAVRKLRAGDFSEVDENVKCFAKCFQERLGYLKNGSVDEDAVNKSLGPLAGEEKVKAVQAKCNGATGSNDCDAALERYKCYYGENMKILA

>CstyOBP20

MKFLVVFAVLVLAVTNIRAQLTKVEAIAIATGCKEETGASDADFEAMLKHQPADSKEGKCLRACTFKKLG

VIGDDGKMLKDAAVELSKSFVKDEEKKKLVAGAIEACNDLKVSADHCEAAEEYGQCLKKEFDSKGISAAD

DLL

>CstyOBP16

MKVYITLAVVCLIASAVAHHELSEEEKAKIKVHFQECVKQENVSEEEATKLRNKDFANATPGMKCFGACF

FEKVGILKDNVVQEDVVLAKLVPHYGEENVKKVMEKCKNEKGADRCETGFKIYECVEKAKAELGH

>CstyOBP15

MKFLIVSGFLILAACSIRAELTKEEAIAIATDCKEEAGASDADFEAMVTHQPAISPEGKCMHACALKKFG

VLSDEGKLLKDAALELSESLIKNEEKKALIAEIVETCDQLEVSDDHCEAAEEYGQCWRNEFEAKGISPDE

DL

>CstyOBP14

MKKYILIEAAASVVVAVLLLMPILSFAQKPRRDENYPPPDFLKKFSIIHDVCVEKTGVTEAAIKEFSDGD

IHEDPALKCYMNCIFHEVNAVDDDGEVHYDKMRRLIPDSLIEFIKPIIDACETHIPKGGTQCDRAWSWHV

CFKESDPVHYFLL

>CstyOBP12

MKVFFVILVLTVAALADHHEGHDHHDHDHHVHHESHDYVVKHHDDLVKFRDECSTKLKISPELMEKYKSW

EYPDDEATHCYMKCIFEHFGFFDEHKGFDVHKIHHQLVGEHVTVDHNDETHHKIEHCADKNTQGSDACTW

AYRGGMCFIRSNLQLVKGSVHKH

>CstyOBP11

MYRLQFYLIYFSLIFLGSIETLQKAKDNGDTYIVKEEKFLKDIKQSTEPASLTTTSSPGNSNIVNLDDMV

STCNTSFSIPMQYYVKFNKTGELPDLVDKTGMCFIRCVFEKSGFIENWKLNAEKIRANIWPAMGDTIEVC

EKEEAKEKNACVRTYAIAKCLTLRSLVDARNNPV

>CstyOBP10

MAFKAFYRLQLFHTILIVLSLMATWTRAQQPRRDSDWPPPSILKMAKPFHDICVEKTAVTEEAIKEFSDG

QIHEDEALKCYMNCLFHEFEVVDDNGDVHLETLFKNIPGSIRDLLMKASENCVHPEGDTLCHKAWWFHQC

WKKADPVHYFLI

>CstyOBP9

MKFWIIATIFLVITILMVSNNNAFEIPEHLKKHAKKLHKRCQNQTETPEDVIRESLTGVLPKNKNFECYV

QCLFDIIGIMDENNIIRIDLLMQVLPDEMHSTLTRLAEVCGTKEGNDKCSVAYNTLQCYVDNNPLMIKNN

LEFLFD

>CstyOBP8

MKLFILCMALVLAAVEVKSDKKEELRNVEKVCREENNITEEELQSAVKSGFKEEPREALKCYMKCILEKL

GQWKNGAFEENVAKKFLQDIPALKDHQDIIEKTLNECKIQKGVNECDTAYLISKCFMERNPRVM

>CstyOBP7

MKASIAILIALFALVSAEYKLRNQEDLMKARKECMEAKKVSPELIEKYKKFDFPDDEVTRCYIECIFEKF

QLFDPKDGFKNENLITQLGHGKENKDEVKADVEKCADKNEQKTDSCAWAFRGFKCFISKNLPLVMESLKK

N

>CstyOBP6

MAKLFVTLAILSVFGGVLVKGFDKKAAVTAFMAKMDDCKAEVGAKDSDVEELVGKKPSSTMEGKCLRSCL

MKKYEVMDNTGKFVKDVAVTHAHKYTDGSEDRMKIAHEIIDACSSIEVADDHCEAAEQYGKCFKEQAVAH

NIKDDFDY

>CstyOBP5

MQFLFVFTFLILAACNIKAELSKDEATAIALACKEEAGASDADFEAMIKHDPTETPEDKCMRACVFKKFG

VMSHDGKMIKDAALELSKSLMKDAEKREIVVGVIDTCEKLVVSDDHCEAAAEYRHCWRKELAGKGVSSAE

DLF

>CstyOBP4

MNLLPKLLICFTIIALSYADEDGMSVKDIAEALISFGEDCDPKPEEEHIIEVVKNVPDAQYTSKCFRHCL

MVQFEIIQEGSQVMDKEKTLDMMGSMFSDRKDDLGEIIDECNSKNEAIAEKCENAHAHGMCMLDLMKERG

FDIPDLKDE

>CstyOBP3

MILKQYLVFLIGAILCLCKVSDVIGGATEEQMWAAGNLMRDVCAPKFPKVTKEIADGIREGNLPNEKDPK

CYVNCILEMMQTMKKGKFLYEGSLKQVEILMPDHFKEEYRTGLAKCKDAAVGIKNNCDAAYAIFTCLRAE

ITRFVFP

>CstyOBP2

MKLFSKLIILKFAILCLSCIQLTSAITMEQFEQSLDMMRNGCAPKFKVSLEQLDKLRNGYFDESSSELKC

YTKCVAQLAGTVTKKGDFSISKATAQIPIILPPEIQETAKAALASCKEIQKDYKESCDRIFYVTKCVRDF

APDIFKFP

>EbalOBP6

MKKYTIVFFVVLINTLSNAATVPDRDALLKFVRAAIDDCYEDDAKTIKVEATGAAFESLITSDPNPPRATKCMRFCVMKSHNLYNEDNTLNIKQVQELFKHVYPEIMDETKLNIVGETTEQCVSHSATVEDRCEKSHDIAMCMITKLAQRGIDIKQI

>EbalOBP10

MKFIIFISFAVAIFNTVNAWSSENSSKYIDECRVELKISDDVNQYNLTTGEIPEADIHPDMKCFLNCFMEKLGILKDGIIQEDADGFNHYVGAETAKEMIESCRDETGTSNCDTAFKLHRCFLKHLSYEFYRFLLMQNGGDDELIQKLLMAVV

>EbalOBP12

MKFFIAIVLLAALASAEYTIKKREDLMKYRSECVEKLSVPTELSEKYKKWDFPDDATTHCYMKCILEKFELFDEEKGFSVENIHNQMVGGHHADHTDDTHAKIDKCAKEATGTDACERAYKGSMCFIRENLQLVQKSVHAHEHDHSAHHH

>EbalOBP13

MLSVRFLIVCVLSVGLIGIQHIDGIPMECTSTKSASSMDLKEVMDTCNSSFTIPMDYIIEFNTTGILPDETDKTGMCYIRCAFEKLGLIKDWKLDKPLLQNTMWPATGDSVEVCEQEGKSESNACVRTYAIAKCLMIRAIVDARDKQVI

>EbalOBP29

MKSVIAIIFALTCICFVIQECKAHDLKTHMREVSQMCQNREQTTDEIVEKIRSGEYDANDVERLAKCHVKCMMEGFGAMENGSLSEKAFVHKLAPHIGEAKAREMFDFCKDESGGEDECDKPFKIYLCLKKLSDIFKY

>EbalOBP23

MKIFIPFLVLICGFCGFVLGQQPRRDDEWPPKGILAMIKPITESCVKKTGVTQEAIREFSDGEIHEDEALKCYMNCIFHEFEVVDENGDVHLETLFRSVPDSIREVLLNMSKNCIHPEGDTLCHKAWWFHQCWKKADPVHYFLP

>EbalOBP30

MKFFIIFIAFIVVFHNTVKAFSEEGPKIYMAECRDKFNITSNLAHYTLIIGEITEDTATEDEKCYLNCFLEKLGVLSDGELQEDAQGLKDYIGNKSAKKAIERCREEFGFHKCETAIKLHRCFLGVRNYAITQIYSS

>EbalOBP27

MKFLILVAAIALFDIVSAVEDCQFEKLKLTDDQIRKLERGQLTDASEDIKCFIECDMEKAGLFKNGKLQEDAAMEKFTAKVGKENAEKILNSCRGEKGSTNCETAFRLSNCFSPALIELLQKKV

>EbalOBP22

MSFLLKLILKIGTFALLVDYAMAGATEEQMMSAGKLMRDVCLPKFSKVSAEVADNIGKGIMPDEKDVKCYINCILEMMQTIKKGKFLYESSLKQVDILMPDHYKEEYFNGLAKCKDAANGIKNNCDSAYALLKCLHAAIPRFMFP

>EbalOBP31

MDKYMIFAFLFFGNFSLYKAIEIPEHLKAHAKRLHDKCQKEIGVDEELIAQSRNGNLPNDRKLECYIHCLFQKTGLIDENNIIHLEHMIEILPTEMQEIIERLIDSCGTKHGADPCETAYLTVKCYFDADPENAILI

>EbalOBP39

MKFFAAILIFSTICLVIEAEQPDQKFVGFFMKLADTCKQKVGPPEEDVKKLFEGAFDKGGASKDVQCYIKCMMEEFEAADDDTQKDLKANMNQEAANECNLGKTGDDCEQAYKTFMCVKENGDKIFKKE

>EbalOBP26

MRFTIAIILIFATLASTSPRNRINNSDVNKYLQECGAELKIPTSEMNKYKAPEDVPDDRIGQCFTKCMFEKFGIFDKENGYKLEPIFKLMSENNHPLVGDIEFIAVIEKCVKESNLIQNACERAYHGSKCLYSDNFKKKNVA

>EbalOBP40

MKFFIIIAFIAIASIANADIPKESDEQFNVAIKGCLEEHKIKEEDYVKLRNGEVANPDENMQCMVNCVMEKTGVLVKGKLQEEVASKIIEKKLGAEEAKAVVNKCKNEPGSGCEVAMNMHLCFLKNKAY

>EbalOBP34

MKFLIIVVVIFLVDIISALEDCNIKFNLTDEEITKLKRAQLTDPSEDIKCLIECEMEEAGLIKNGELQEDVVIEKFGKENANKILESCRGEKGSTNCDTAFRLHNCFTRTRRRAALLEVLSRKV

>EbalOBP35

MKLNLLKMKFLIIVAVIFLVDIVSALEDCNTKFNLTDEEITKLKRAQLTDPSEDMKCLIECEMEEAGLIKNGELQEDVVIEKFGKENANKILESCRGEKGSTNCDTAFRLHNCFTRTRRRAALLEVLSRKV

>EbalOBP19

MKNIVSLLIALGSWQFIEAMTESEAIGALEGFGDKCEPKPNEDDYKNIVRNTEDVPQSTKCFRLCLMEQMDLIVDNCKLDGEKLTDLLTMAFDGKEEETAEIANHCNLKVECTDKCDAAHAHSMCILNQMKIKKWPLPELEEDSKQ

>EbalOBP20

MKYFLVFFVFGVLCGASVLCQKVEPRRDETYPPPELLELLRPVHDVCVQKTGVTEEAIKEFSDGDVHEDEALKCYMYCVFDQTNVLHADGEVHLEKLHDMLPDSMHDIALHMGKRCLYPKGDTTCERAFWLHKCWKQADPKHYFII

>EbalOBP36

MKLLIVLAFIAVFAAVNAEIPKEDDEEHNKIIAECRQKFKMTDEEYTKLRHDEVAKPNEDMQCFVNCFMESAGMIKDGKLQHDVATAIISKKVGEEKAKTILETCHGEQGSTNCETAYKLHKCLYKNKAY

>EbalOBP33

MKFFLAIAFVTIVVAVNAANIPDAHKEKVMQIVAECQKEMDISAETLAKIKAGEPFGADEKTKCFANCFQEKAGILKDGVFQEEAIVAKFSESFGADKTKSIVDACRGEATGKDNCEKAYNLHACFKKNNAY

>EbalOBP41

MKFFIVVILVAICGSALCGENDWEDNLKQCADKNKVKLDELKDALNAKIKEADVTNDMKCAFLCTAEKEKIIQNGAFQPQVYAEAVAATADKDQVAKITSECNLKGKDDCETAYKVGVCIAKFHNK

>EbalOBP25

MKYLAVGILFTIFAFTSAQEYKVKTQADLVNIRKQCVDLKKITPEQVEKYKKFEFTDDEKTRCYIECIFDKFGLFNAKDGFKVDNLVKQLGQNRNQTEVKAEIQKCVDKNEQRSDSCSWVFRGFKCFISKNLPLVQQSLKAN

>EbalOBP38

MQYFIIVILVSICGSALCTEAEWDENKKYCSEKLNVNLDEARDAVRGRTKEADVTKDIKCHFLCMGERQKIIKDGVFQPQVFKQILSAVDDKVLLTKATEECSKKGTDDCDTGFKVATCISRNDLRKYM

>EbalOBP32

MKFVLITIFAFACFAVEAVTSEDDAALTHAAIEKCKGPAGVTDEEIEQLKTDKFHDGGADKHIMCFVKCVLDEFDALDGDVLKEGVFLDYFEPHLGRPKAKEYYDLCSGEAGDDECETPFKIIMCLGKSDDIFKI

>EbalOBP24

MKLISFIVLCTLAAAVKGAISKEAVETVRTLSNECLKEIGATDAVFDEMIKNLPATNMEVKCLRACLMKKVNVLSPDGKLNKENALKMAEMHTEGDAEKMKIAHAVADACEAISIPDDHCEAAEAYKMCILSEAKKHGVNGLI

>EbalOBP28

MKYLIFISFVIALFDAAKAASTEQSTKYIAECRAEFKITDDVKHYNLTTGAIPEADATEDMKCYLNCFMEKLGVLKDGKIQEDAQEFKNYVGEEHAKEEIESCRGETGSSKCETAFKLHQCFLKHLDYLVLQVFSLSE

>EbalOBP17

MFKDSTIWLASSVVFSALFSSTFSVTMKQFEASLEMMRSGCAPKFKVTTEQLDDMRNGKFIENNMDIKCYTKCIGQLAGTLTKKGEFSIQKALAQIPIILPPEMQDSAKSSLEKCKDIQKGYTDSCDKVFYVTKCVHDADPPSFKFP

>McorOBP8

MKTCISIIFIALLVNWTSADTLTLESKLKTYEKYREECLNSFQIPESELEKYKSNTFEDSVYDNAIGHCFIKCFLEKVGVFKNDTGFQENHIFHETIGGFIPDQSRQFLTKIEECAKNANQETNTCKRAYVGVSCIVNHLKTVFKPFEDHQ

>McorOBP9

MISVKVLMVCVLSGGLFGFQLLLADGIPMQCTESPSTMDLKEVMDICNASFSIPMEYLTEFNTTGVLPDEVDKTGMCYIRCAFEKLGLIKDWKIDKPLLQKTMWPATGDSVEVCEKEGKSEPNACVRTYAIAKCLMIRAIVDARDKQVV

>McorOBP14

MFKDSTIWLASVVFSALFSSTFSVTMKQFEASLEMMRSGCAPKFKVTTEQLDDMRNGKFIENNMDIKCYTKCIGQLAGTLTKKGEFSIQKALAQIPIILPPEMQESAKSSLEKCKDIQKGYTDSCDKVFYVTKCVHDADPPSFKFP

>McorOBP16

MSCLLKFILKIGIFACFVDYSIAGATEDQMMSAGKLMRDVCLPKFSKVSPEVADNIGKGIMPDEKDVKCYINCILEMMQTIKKGKFLYESSLKQVDILMPDHYKEEYFNGLAQCKDAANGIKNNCDSAYALLKCLHAAIPRFMFP

>McorOBP17

MKAISVFVLCTLAVAGKGSAPIEAIDLLRSLSAECLQELGASEAVFDEMMKNLPATSMDIKCLRACIMKQVSVLTPEGKLHKDNALKLVEMHVNGDAEKMKIARAVGDACEGIAVPDDHCEAAELYKMCMVDEAKKHGINEIL

>McorOBP18

MKYLAIGILFTIFAFTTAQEYKIKTQADLANIRKQCVELKKITPEQVEKYKKFEFTDDEKTRCYIECIFDKFGLFNAKDGFKIENLVKQLGQNRNQTEVRAEIQKCVDKNEQRSDSCSWVFRGFKCFISKNLPLVQQSLKAN

>McorOBP19

MKHGAVIRVFSLICLVSLVSANEKAHEDIINYIKGVIEGCKTKMNTPDDIFEDIWTGKYDKGGANEVAMCHINCMMKEFGALKDGNVEAFETFKEHIHETAAQEVLEKCQQETGETDCETGYKIYMCMKANSEKMFKPQ

>McorOBP20

MDTKFMVFALLFFVNLTLNEAIEIPEHLKAHAKRLHDRCQKEIGVDEALIAQSNNGNLPNDRKLQCYIHCLFQKTGLIDENNIIHLEHMIEILPTEMQEIIERLISSCGTKHGADPCETAYLTVKCYFDADPENSMLI

>McorOBP22

MKFPLIVAFIALMVISIVIADHDYPNETIKEELDKKAAECRSKFKVTDEDYKKMLVEFAEPSETMKCLSNCLLEVVGILKNGILQPDIAIEVFKSGYREEKAIRIVEACRNEVGIGKCGTANKLDMCFLNNQYLKS

>McorOBP24

MKFLIAIVFVATIVAVIAAAAGFEIPEDEMTRAMGIVGNCRDEITITDEEFEKLKDGENFAASENAKCLTSCIQEEAGITKDGVFQADAVLAKFAPLVGEEEIKKVIEACKNESGEGKCETSYKLHQCFKKLDAY

>McorOBP25

MKFVLLVAIVVACIGGQVWASDDDAALTHAAVEKCKGEISITQEELEQLNGGEFDDGNGHKNVMCFVKCVLDEFDALDGDVLKPDVFHDYFEPHLGAEKTKEFYDGCKGEAGDEECETPFKIVMCLRRSDDIFKF

>McorOBP27

MKFFLAIAFVTIVVAVNAANINIPDAHKEKVMQIVAECQKEMDISAETLAKIKAGEPFGADEKTKCFANCFQEKAGILKDGVFQEEAIVAKFSESFGADKTKSIVDACRGEATGKDNCEKAYNLHACFKKNNAY

>McorOBP28

MKFLIVFALIAIVSVVSAADADVKEQNQRVEEHVTKCRSKHPIKDEQLALLKDGKVTEGSDDERCFVNCFMEESGIMVDGKIQKEKAIKAFSVRIGEEKAIEAFEKCQSEVGSAKCETALKMHNCFHAQGVY

>McorOBP29

MREVSQMCQNREQTTDEIVEKIRSGEYDANDVERLAKCHVKCMMEGFGAMENGSLSEKAFVHKLAPHIGEAKAREMFDFCKDESGGEDECDKPFKIYLCLKKLSDIFKY

>McorOBP30

MKSILVIALIATFAAESVLAGLTPEQAMEHVTFCKKELNLNDADFKQLVQAKTFADVNEKSKCFFNCFQESEGTLIDGVLQEEKVMDLFIGTVGEKKAREIYDICKKEKGAEKCETAFKLQICYRENGIF

>McorOBP31

MKLLIVLAFIAVFAAVNAEIPKEDDEEHNKIIAECRQKFKMTDEEYTKLRHDEVAKPNEDMQCFVNCFMESAGMIKDGKLQHDVATAIISKKVGEEKAKTILETCHGEQGSTNCETAYKLHKCLYKNKAY

>McorOBP32

MKLLIVLAFIAVFAAVNAEIPKEDDEEHNKIIAECRQKFKMTDEEYTKLRHDEVAKPNEDMQCFVNCFMESAGMIKDGKLQHDVATAIITKKVGEEKAKTILETCHGEQGSTNCETAYKLHKCLYKNKAY

>McorOBP33

MKFFIIIAFIAIASIANADIPKESDEQFNVAIKGCLEEHKIKEEDYVKLRNGEVANPDENMQCMVNCVMEKTGVLVKGKLQEEVALKVIEKKLGAEEAKAVVNKCKNEPGSGCEVAMNMHLCFLKNKAY

>McorOBP12

MKYFLVLFVIGVLCGANVLCQKVEPRRDETYPPPELLELLRPVHDVCVQKTGVTEEAIKEFSDGDVHEDEALKCYMYCVFDQTNVLHADGEVHLEKLHDMLPDSMHDIALHMGKRCLYPKGDTTCERAFWLHKCWKQADPKHYFII

>McorOBP15

MKIFISFSVILICGFCGSIQGQQPRRDDEWPPKGILAMIKPITESCVKKTGVTQEAIREFSDGEIHEDEALKCYMNCIFHEFEVVDENGDVHLETLFRTVPDSIREVLLNMSKNCIHPEGDTLCHKAWWFHQCWKKADPVHYFLP

>McorOBP23

MKFLILVAAIALFDIVSAVEDCQFEKLKLTDDQIRKLERGQLTDASEDIKCFIECDMEKAGLFKNGKLQEDAAMEKFTAKVGKENAEKILNSCRGETGSTNCETAFRLSNCFSPALIELLQKKV

>McorOBP34

MKTFVIALIVLMIQEIIAGPEDVICRQKIGITFEESSDFLQRAKIPEIRDQMDQKYKCFVLCLMEEMNILDGCSYQLELGKQRVSEMGLAKLIPILDSCKDSSVGSEPCDCGYNVFKCVLDGMMAMEEQ

>DmelOBP99b

MKVLIVLLLGLAFVLADHHHHHHDYVVKTHEDLTNYRTQCVEKVHASEELVEKYKKWQYPDDAVTHCYLECIFQKFGFYDTEHGFDVHKIHIQLAGPGVEVHESDEVHQKIAHCAETHSKEGDSCSKAYHAGMCFMNSNLQLVQHSVKV

>DmelOBP44a

MKNAVAILLCALLGLASASDYKLRTAEDLQSARKECAASSKVTEALIAKYKTFDYPDDDITRNYIQCIFVKFDLFDEAKGFKVENLVAQLGQGKEDKAALKADIEKCADKNEQKSPANEWAFRGFKCFLGKNLPLVQAAVQKN

>DmelOBP8a

MMRRSQIGLLSRLLLLLLVVELTPPAIPVPMRSSPQSLALLRARDQCGRELTAAQRLQLDRMQFEDAAHVRHYLHCFWSRLQLWLDETGFQAQRIVQSFGGERRLNVEQALPAINGCNAKTSSRGSGAQTVVDWCFRAFVCVLATPVGEWYKRHMSDVINGNA

>DmelOBP99d

MNHLRLEIICWSCLLIAMAVSTEAASVWKLPTAQMVYEDLEKCRQESQEEDAATLRCLVKKLGLWTDESGYNARRIAKIFAGHNQMEELMLVVEHCNRMEQDTSHLDDWAFLAYRCATSGQFGHWVKDFMSQKEVER

>DmelOBP99c

MLKYLIVALALCAVAHADDWTPKTGEEIRKIRVDCLKENPLSNDQISQLKNLIFPNEPDVRQYLTCSAIKLGIFCDQQGYHADRLAKQFKMDLSEEEALQIAQSCVDDNAQKNPTDVWAFRGHQCMMASKIGDKVRAFVKAKAEEAKKKAA

>DmelOBP99a

MKVFVAICVLIGLASADYVVKNRHDMLAYRDECVKELAVPVDLVEKYQKWEYPNDAKTQCYIKCVFTKWGLFDVQSGFNVENIHQQLVGNHADHNEAFHASLAACVDKNEQGSNACEWAYRGATCLLKENLAQIQKSLAPKA

>DmelOBP83g

MQSQSLLLIVAAVATFLVAQTTAKFLLKDHADAEKAFEECREDYYVPDDIYEKYLNYEFPAHRRTSCFVKCFLEKLELFSEKKGFDERAMIAQFTSKSSKDLSTVQHGLEKCIDHNEAESDVCTWANRVFSCWLPINRHVVRKVFA

>EbalOBP5

MKIYIVLLLIALTSAAEWKIKTNKDWDEIEDTCFERHKNLVEQRGNSKSKDLTKPEFELVLCVFREGEVWSDSKGFSTDRLMMVMDTVATRDNINKKFLRDSLENCADDNSEGSSPLDWGYRYYKCFKDNEVLYETMRKARFIQPDVVGKQ

>EbalOBP11

MKICIILALIAFSSAAEWKIKTLKEWDHFEDICLERYKELIEKHQNDRTEEYPKEAFEILLCVFREVGIWSDSKGFSVDRIMIMMDRIATKENVNKQFLRDGLEKCADNNSEGSTPLDWAYRSYNCFKANKVLYETLTKGRFGADQETVNA

>EbalOBP21

MKTFLVVLAFIGAVLAEEWVPKNLNEINEIRRECLVSNPLSVIQLAAMKKFVYPDEEQVRKYLLCDVKKLEIFDEREGWYEDRIVKQFKMRLDEAEATTIVEGCADKNEQKSSADVWVYRGHKCLMASKIGDEIKEFMEYQIAKEA

>EbalOBP14

MKFFAAVLVLCVASASAAVLKDKTEDEFFTASAACAKKLAVDDSHLAKFQELDYPNDKTTQEFLHCLWTGMDLFNDEIGYNVENIAFLYKDKANSEVLIPILSECNKKEANDSTLSWLYRGFQCIMSSKVGQWFKEDIAKKQAALAASS

>EbalOBP15

MKLFIILALVALTSATKWKPKTYKEWQEVEVKCEEQHKVSPEIKEKAKTARYLPKEQFEKNLCYMRGAELWNDSKGYNVDGMITLIKSIPAEENIDKDSQIDIFMKCIDNNSEGSHPIDWAYRGYKCFRDNGNLYTNLGKGKYYEEPEN

>EbalOBP7

MKIYIILTLIALTLAEEWKLKTVEEWKQIYMTCDQRHQVAEEFYQKSRTEKYPPKEVFEVVLCTMRSMEVWSDTEGFSVDKMMIALDSAATQENVDKKFIRDSLESCADKNTEGSTPLDWAYRCFKCFKDNEKFFKVLREARFFDEQSSDYVDSK

>EbalOBP8

MKIYIILTFLVALTSSAEWKLKTMKEWRQISEACTQRYPVSPEVLEKAKIDKYPPKEMFQAILCTLREIDIWSDTEGLSIDRLMIVLEKAAVEENISKKFLRDSLESCVDKNSEGTTSLDWVYRCFNCFKGNEKLFKVIEEARFYEESEPLNYEW

>EbalOBP9

MKIYIILALVALTSAAEWKRKTMDEWSQIHRACDQRYKVSEELIEKAKVEKYPPKEVFEVALCVLRDSEVWNDATGFSVDRLMISMNTVATRGNINKKFLRDTLESCADNNSDGSTPLDWAYRCFKCFKDNEKFFKVLREARFFDEQSSDYVDSK

>EbalOBP16

MKIYIILALVALTSAKWEPKTHEEWLEIEAKCKEQRKMTPELEERIKNEPYLPKEAFEFNLCCLRSTDLWSDTEGFSLEGMTAILDRIPDEEKIDKDAQRDILKKCIDNNSEGSTPFDWAYRCYKCFKDNGDFLKNMGKAKFHDHKEH

>EbalOBP18

MKSFIILTLIALTTAEWTLKTQDEWLKIREDCNTRFHVPNELIEKTKNDKYPPKEVLANVLCYLKGLEIWNDKDGFLVDRIMIGLENVAKKIENYNAKLLQEGLKSCINQNSEGTTTFDGAQRCFKCFIDNTYLFEALKKTSFYQE

>AfraOBP99b

MKFFIIAFLSLIALVLADHDPDHAGHSDYVVKTNEDLVEARKQCVSKLSIPDDLVEKYRKWEYPDDEKSRCFLKCIFEQFGLYDDEKGFDIHKIHHQLEGDKVDHSGDVHAKIENCAKEGADAADACTRAYRGITCFLKNNLSLVKQSVGSA

>AfraOBP99a

MKYIIAVLLALFALAAAQEYKVRNQDDLLKARKECMEAKKVPADHIEKYKKFEFPDDEVTRCYIECVFNKFQLFSPTEGFKTQNLVAQLGHNKENKDALKADIEKCADKNEQKSDSCTWAYRGFKCFISKNLSLVQESLKKN

>AfraOBP99c

MKYFIIAILAIVALAHAEDEWKVKTAADIKVIRQECIKEFPLSEEDIQKMKNFEYPDEEPVRKYLLCTAKRLGIFCAHEGYHADRVAKQFKMDLDEAEVLAIAQGCADKNEQGSSADVWAYRGHKCLMASKVGEKVKAFIKKRMEEAQKQ

>AfraOBP99d

MPTWRALFIFGAVVLCCVDTRTVDATPTAIAAAAASEEPVRRLRKAHKICAQEISPPATDESDNTQIAGA

YLRCMTNSMGLWTDGKGYNAKRVAKFFSKQRNENEVVVVVDHCNQQHKQADLNLWAFEAYRCATAGRMGT

WLGEYLLSAKI

>AfraOBP8a

MRSTTLAFIVFLFTFDAFSAADFEEKSEDDFLSASERCFERERLPSSYQRRFDNFEYPDEEIVHRYVHCIWKELELWNDRTGFNVEHIAALYRDKANTEVLVPILSDCNRNAQNESKLKWCYDAFKCILNSRVGQWFKEDVGRKLHDTKKANHVD

>AfraOBP83g

MKLQLILLLACVALASAKFQLRTAQDAIIAHEECRDEFRIPEDIYQKYLNYEFPAHKRTNCYVKCFVEKMGLFTEEKGFDEKAIIAQFTAKSTKNLAKISHGLEKCIDHNEHDSDTCTWANRVFSCWISVNRPIVRKTYIAK

>AoblOBP8a

MRSTTLAFIVFLFTFDAFSAADFEEKSEDDFLSASERCFEREHLPSSYQRRFDNFEYPDEEIVHRYVHCIWKKLELWNDRNGFNVEHIAALYRDKANTEVLVPILSDCNRNAQNESKLKWCYDAFKCMLNSRVGQWFKEDVGRKLHDTKKANHVD

>AoblLOBP99b

MKFFIIAFLSLIALVLADHDHDHADHSDYVVKTNEDLVEARKHCVSKLSIPDDLVEKYRKWEYPDDEKSRCFLKCIFEQFGLYDDEKGFDIHKIHHQLEGDKVDHSGDVHAKIENCAKEGADAADACTRAYRGITCFLKNNLSLVKQSVGSA

>AoblOBP99d-2

MPTWRALFIFGAVVLCCVDTRTTDATPTAIAAAASEEPVRRLRKAHKICAQEISPPATDESDNTQIAGAY

LRCMTNSMGLWTDGKGYNAKRVAKFFSKQRNENEVVVVVDHCNQQHKQADLNLWAFEAYRCATAGRMGTW

LGEYLLSAKI

>AoblOBP99d-1

MPTWRALFIFGAVVLCCVDTRTTDATPTDIAAAPSEEPVRRLRKAHKICAQEISPPATDESDNTQIAGAY

LRCMTNSMGLWTDGKGYNAKRVAKFFSKQRNENEVVVVVDHCNQQHKQADLNLWAFEAYRCATAGRMGTW

LGEYLLSAKI

>AoblOBP99a

MKYIIAVLLALFALAAAQEYKVRNQDDLLKARKECMEAKKVPADHIEKYKKFEFPDDEVTRCYIECVFNKFQLFSPTEGFKTQNLVAQLGHNKENKDALKADIEKCADKNEQKSDSCTWAYRGFKCFISKNLSLVQESLKKN

>AoblOBP83g

MKLQLILLLACVALAAAKFQLRTAEDAIIAHEECRDEFHIPEDIYQKYLNYEFPAHKRTNCYVKCFVEKMGLFTEEKGFDEKAIIAQFTAKSSKNLAKVSHGLEKCIDHNEHDSDTCTWANRVFSCWISVNRPIVRRTYIAN

>AoblOBP99c

MKYFIIAILAIVALAHAEDEFTVKTAADIKVIRQECLKEFPLSEEDIQKMKNFEYPDEEPVRKYLLCTAK

KLDIFCEHEGYHPDRVAKQFKMDLDEAEVLAIAQGCADKNEQGSSADVRAYRGHKCLMASKVGEKVKAFI

KKRMEEAQKQ

>CstyOBP27

MKVFVAILALVACVSAEEWTVKTDDQIKEISTECLKEHPLSAEQINKIKNFVYPDEEEVRQYLLCAVVKS

GVFCTHEGYDAGRVAKQIKMDLDEEEVKKAVEDCIAKFPKGDKANDVVVLETHTCLMSSTTGEKLKELLK

KRRETAEKHE

>CstyOBP25

MKVFVAILALVACVSAEEWTPKNGEQIKEVRIECLKEHPLSPEQMNKMKNFEFPNEEAVRKYLLCTAEKM

DIFCSHQGYHADRIAKQFKMDMDEEEVQKLVEDCIAKFPKGDKPNDVVAYEGHSCFMSSAIGDKLKNYIK

KRHEAAQQHE

>AgamOBP69

MEWRYFVVIALICPLIIVETLAVSDCVRHVSESARNTVCDVRQYRVTKGVEADRYVQCFMTALGFADESGSIQRSNVLTALDAVETHDGVYTDAVDVCLSKAKKLPGTERSGSFFSCMLRTESAQNFRDAVELQELRVASKWPEGERFDRSKVQLMMRELNSQLRC

>AgamOBP70

MIRQVITSYFLTVCLLALVQGETVQDCENKLPTSLKSRLCEIRRYEIIEGPEMDKHIHCVMRALDFVYEDGRGDYHKLYDPLNIIELDKRHDVNLEKCIGECVQVPTSERAHVFYKCLLKSTTGRTFKKVFDLMELKQAGKVPQHQRYTAEFVQIMKDYDKALNC

>AgamOBP71

MFKKLLLSVGLVWCLISLGQARKESTVEECEKNIGDSLKDRVCELRQYTPVSSDDMDKHMQCVLEVVGFVDGNGEVKESVLLELLQRVDSGVNHAANMKKCVTEASTSGSDKKANTFYTCFLGTSSLAGFKNAVDYNELLKAGKMQTSDPFDMNRVAALIKEIDDGLC

>AgamOBP72

MFGKLLPCAILVWCLFSLGQARQEETVEECERNIPASLKGRVCELRQYTPVQGKDMDSHMQCVLEVLGFVEDNGELVFQELLGVLKMVDPDGDHASSMKKCNAEAEKVDTSSKANTFYTCFLGTSSAQAFKYAVDYVELLRAGKLDMGTTFNAGQVSALMKQIDDGLCN

>AgamOBP73

MFNKLHFVSLLACGLFVIAQANTVKKCEKKMPASLKSQLCEIRKYKLLDTPDMDKHMDCVMKALDFVRPDGTGDYHKLIKPLNAIEKDRKHDFNLEKCGGQTQHLPVGKRANAYYKCLVESTSGEAFKKVFDTVELVKAKKLPALSQYSSVVDKMMKKIDDKICN

>McorOBP6

MKTSIIVFLAFIAITSAEEWKLKTMEEWSQIHKTCDERFPVSAELIEKAKVEKYPPKEVFEVVLCILRGVEVWDDTKGFSTDRIMFGLENVAKRENLSKQFLRDGIEHCKDSNSEGSSTLDWAYRWFKCFKDNEPLFKAIREVKLYKERERKIQE

>McorOBP7

MKFFIILVTLVALTAAKYELKSREEWFNIQDYCDERFKVSPEFREKHKDDKYVPKEIFRTILCYLRGLELWNDSEGFSIDKLMVGLESVKDNIKNYNAVILRQSLAQCTSDKNTEGIAPFDWAYRCFKCFTDNRYIMEAFKVATFYKEDKKDADE

>McorOBP10

MKFLIAVVVLAALASAEYQVKTREDLTKFRDECVAKLGTPQAELEKYKKWNFPDDETAHSYLHCILKKFELYDDEKGFNVEDIHKQMVGGSHADHSDDTHAKIENCAKEANAAEANVRAYRGALCFMREHLHLVQKSVHAHEHDHSQH

>McorOBP11

MKLFAAVFALCMVALASSAAFQDKTEDDFFKASAACAEKLKIPASLLEKLQQFEYPDEELVHEDIKCVFSTLELFNDKTGYNVEHVADLYKDKANAEELIPILSNCNKNPTNEPAAKWAYKGFQCIMASKVGQWFKDDITKQAAAKA

>McorOBP13

MKTFLVIFAIIAAVVADEWVPKKFEEIKGIRAECLASNPLSTEQVDSLKAFIYPDEEPVRKYIQCCSEKLEIFCEHEGYHVDRIVKQFKLKMDEAEATAIVEKCVDKNEQKSSADVWVFRGHKCLMGSKIGDGIKEFVQKAMAKKA

>DmelOBP93a

MYVYNLLFVVIVFSYCAKSFNYTSCDHAKQPKFLSSCCDVQKNDKAINSCRKSLLGNNSTNSNGEVRNLKSDKVALHACIAECSFRTNGFLLSNGTVNTQALQKSYQQRYKNDPNMSQLMLKSLNSCTDYARKRVQEFQWMPKKGDCDFYPATLLACVMEKVYINCPTSKWKNTSDCTAMWKYLVACDDVASNKKK

>DmelOBP50b

MSSVLHLLGFLWLPLLVYSVSNDMGGLQKCTELLNTHKLVYCCGKSFLDKFPFVGSNCTPFWDDYGPCRYECLYRHWDLLDQDNKIKKPELYLMITSLYSPLNGYDKYGAAFKAAHETCEALGSRHADFLLLYSNQVADKMGMASSTCLPYAMLHAQCTMVYLTANCPRENWIDDPKCNSLQKLLSSCTKKLDEKTNALKGKDEELTDNGCGHIDSEGSNLLMACFLTLMIAKFISDH

>DmelOBP50d

MLHKLTWVLIFIPAFRAADPICSQRPDVTALRNCCKLPNLDFSSFNSKCSQYLVNGVHISPCSFECIFRAANALNGTHLVMENIEKMMKTILGSDEFVHVYLDGFRSCGNQEKVLIKAMKRRRVPITGKCGSMAIMYGLCAHRYVYRNCPESVWSKSATCNEAREYSIRCDDM

>DmelOBP50a

MRTGRILVALIFLGLIIPFRAAKCRAAPKSVQNVHVCCSAPLPNWGVFNRECHKSAIQASCRLDCDFNASSVLQGNRLIQAKVRPMLERAFSNEPTIDAYESNFAKCSTVVRSKYQELSPLSRQSDACDRHALFYSLCAYARLIFTCPDKMWQRNNRMCQEAKAYAKKCPWPALKMFMRNT

>DmelOBP50e

MHKYIICFGFLLIILECSLASFNCSAPPNFNNFDINTCCRTPELDMGDVPQKCHKYVSGLKSANSKYPSYAHLCYPDCIYRETGAMVNGKIKVNRVKQYLEEHVHRRDQEIVSHIVQSFESCLSNVKGHMKSLNIESYKVLPHGCSPFAGIIYSCVNAETFLNCPQQMWKNEKPCNLAKQFAEQCNPLPHVPLPSS

>DmelOBP46a

MCSQLFAFLLLLLTAFVTGRSTPPALDEDCELNSVDTMHDFCCDLHDESPQFSDCQMEWHEKIPYETDEEEQTYMFCTAECSFNSTNFLGRDRRSLNLNEVKEHLESDLVNDADIKLLYDTYVKCDKHALSLMPHKGVKQLSKRLSRLGCHPYPGLVLECVANEMILHCPTKRFRQTAQCEETRNHLKQCMQYLKYKS

>DmelOBP58b

MLRIGFVICVIISLRLNGLVAVRVHCRHMERIHEENIHHCCKHQDGHDDVTESCAKQTNFRLPSPNEEAIVDVTVDQAMVGTCWAKCVFDHYNLMENNTLDMDKVRSYYKRYHQTDPEYATEMLNAYEKCHTQSEEATEKFLSLPIVRAFSTAKFCKPTSSIIMSCVIYNFFHNCPASRWSNTTECVETLAFARKCKDVLTTM

>DmelOBP58c

MKCTILLSFFSLIWFAGGIKIDCENTEAINEDHIHYCCKHPDGHNDLIEGCARETNFTLPNQNEEALVDITADRAIRGTCFGKCVFSKLNLMKDNNLDMDAVRSLFTERFPDDPEYAKEMINAFDHCHGKSEENTSMFLSKPLFKQMSKQFCDPKSSVVLACVIRQFFHNCPADRWSKTKECEDTLAFSKKCQDSLATL

>DmelOBP58d

MVNIVCYWTFLILVAVSKAQDNEETTAVAISSGDLTEDKCNTSRAGCCSELYIGEEEDLVKCFVIHSPKLPVDGDADIGKTLRFLSCFVECLYKQKKYIGKSDTINMKMVKLDAEKTFVDRPKEKDYHIAMFEFCRKDAVGVYNLLKASPGAKVLLKGACRPYLLMVFMCISDYHQKHECPYFRWEGTAKAGTKDMCENAKAECYQIDGITLPTKSPA

>DmelOBP49a

MLSKSQLLLLVVGFCLNAAVSADVDCSKRPSFVNPKTCCPMPDFVTAELKQKCIKFDMTPPPPPDGEASGSFESKRRHHHPHPPPCFFSCIFNETGIYQNRKLDEAKLNAYLQEVFEDSSDLQTTATQAFTTCATKVADFEANLPPRPAPSPPPGFPMCPHDAGHLMGCVFRNMMKNCPDSIRNDSQQCTDMKEFFTKCKPPRGPPPSAEDM

>DmelOBP47b

MSPSQLLVIFASLALNTRLVFGQATIDCQRPPQLVDPALCCKDGGRDQVAEQCAQRILGTANGQKAGGPPSLDTAACLAECILTSSKYIDEPQKLNLANIRSDLSAKFSNDTLYVETMTMAFSKCEPQSQRRLAMIMQQQQQVQQQKTQQQQPRCSPFSAIVLGCTYMEYFKNCPDHRWTPNAQCTLAKAYVTQCGLGA

>DmelOBP50c

MARHIALLICSLLAMAGCDPIDVDCTRRQDFNIVKDCCVYPTFRFDQFKSQCGKYMPVGAPRISPCLYECIFNKTNTVVDGAIHPDNARLMLEKLFGNQDFEEAYFNGLMGCSDSVQEMISNRRSRPQRKTEQCSPFSLFYGICAQRYVFNHCPSSSWSGTESCEMARLQNMNCSKPSRGSSHRL

>DmelOBP59a

MKQLIFLLICLSCGTCSIYALKCRSQEGLSEAELKRTVRNCMHRQDEDEDRGRGGQGRQGNGYEYGYGMDHDQEEQDRNPGNRGGYGNRRQRGLRQSDGRNHTSNDGGQCVAQCFFEEMNMVDGNGMPDRRKVSYLLTKDLRDRELRNFFTDTVQQCFRYLESNGRGRHHKCSAARELVKCMSEYAKAQCEDWEEHGNMLFN

>DmelOBP85a

MSPGSVVFSMFLLLRYFYLFNCEINCKLERKRAADLSIKCCQLTRPSLDKGNSECRKSLNLPAHRKFNFAELYTINMCIEECNFIGCGYIEIDPPFRLDLANIRTNLQTIAPQPQNESIPFLVDAYRKCELFRSSHGRRFTLHLPDIEFIEEPCNPFALQITICVRIHAMQKCPSEFYVDSDECRLAREYFTQCVGDIETNLA

>EbalOBP1

MLKLIVCFSVLFVVSFVTADYDFNDSDFNELIFQDLTTEIEDKPLRVRRGADIESASADNPKDSHRKKAKEEKDAHCCSGEKGNPQEIEAAKEVKNKCIAEIRGESGTDDELGYDPLSCEGVQAMREKTICAAECIVKKLDLLDVNGQFKRDALLNHTRKLIGESKWKTPVLEEYLDGCLSSLKNSTTTSTEVEKEKSEKQSCNTAPLELHHCMWKKFVEGCPVEQQIDSKKCRKVRERLTKGDTSYAEKTFKKLFKHRH

>EbalOBP3

MSNYLNLRKNNMNRFGVLNLVIAIGLVALSVTDAIDCNNKPDFKAAKDCCPFEGFATSEIKESCKSLMSDDDGPPADGGGPGGKGHHGPKHRNSCYHQCIMNATEMVNFETMTVDEAKMKAYLPKALSGTPDFVQPVQDAIVKCAEKGKEMKARHANDDHPHPSPPPGSCKPCASMLMHCVKMETMINCPTSTWKNDEACNNLREFMMVCKPKGPPPPK

>EbalOBP4

MDHYGRNSLKTIICIVLLSVIINNILAVNIDCNRPPPLVDPQMCCTDGGRDEVSEKCAKRFDISDTHSQARMNIETATCLAECVLTESNYLIGQDLNIAAIQADLQEKFPQDPGYVEAMIKSYQKCTPIAQRKLEELRRSPLGSIAFQRKCSPFSGIILGCTYTEYFHNCPAKHWNASEQCEIAKAFLQKCSVF

>McorOBP2

MLRLSFCFSFFIITLVTANYDFHDPDFNEMLFQDITTEIKDITLRYRRSATDNTACECQEDQPKEEKYDVHCCRGQKPKKSEGLKKAREAKKKCFAEIRGDSDPDADVGFSYDPLTCEGIQAMREKTVCAAECIVKKLDLLDDSGAFKRDALLNHTLSTLVGEGRWNAKMMETYVDGCLNELKTVGNEKSKNVKPSCNPLPLEYHHCIWKKLVEGCPVESQMDTKKCQKIRERLTKGDTSHAQKFYKKLFKN

>McorOBP3

MKEVKKECIAQIRGASAPSYDPFDCEQMKLLKEQSVCTAECIAKRFNLVDEHGDMKRESVLVNLRAKIGDNNAWKSEAVEGFVDKCLAELKASKEKSAEAAEAAAALKKESQPIEKESRDEKVGCNPCPLEFSHCIWREVVQGCPAESQIDSGKCKKIREGLAKGDKSFLNKHFLHHFSAHSEDKKSWD

>McorOBP4

MNRFGVLNLIVAIGLVATSVTDAIDCNSRPDFKNARECCPHEDFSGNEVIAKCKDYLNADDNSPPGGHGPGHKKMHFNTCYNECIMNETGLVDFETMKVDEAKTKTFLTDLLKEKPDFVQVVTDAILKCADHVKEMREKHANDPKPTLPPGGCKPCAAMFMHCVKKETTINCPTSAWKNDETCNNLREFMMVCKPPNHRGPPQ

>CstyOBP23

MFKSVISLILLSASLQCYTAAVIDCQRPPQLVDPAKCCLDGGRDEVTEVCAQRMGITGGPSDAPPTVETA

TCLAECILSESKYMQKPETLELSLIKSDLQSKFANDTIYAQTMAEAFQKCQPSAQRKMKAFKQIPLGNVA

LQRGCSPFAGMVLGCTYMEYFKNCPAHRWTDNEECSLAKQFVTQCSLGA

>AgamOBP48

MGQRQRVVVQLALCFLTFGALLQAGVLAGDNPCAAGPPVDTNPAECCPTPMLVDGTIMMDCYKKYGEQTKKQLQMDGIPRGCCIAECAMNATNMYADGMLKRDDLSKMFMDAVKDKPEWMSLVRDATNACFELAEKKMDEIEAGAKLEPSFEGEKICHPISGTILRCMGMMMFAQCPASVFNVNENCNKLREYGSICPMI

>AgamOBP49

MEWNWTFLFRSFLLLTLHLLPQSVADDCIDMDLHSMEVARCCRYEPISTEEVAEKCYQELAPNIPPNSSDFPVCFIDCSYRQMGYITNEANEIDQSKYGQFLAGFDTAYKIAVERAVAACATVQEDIRRDVANVPSKCNAFALLFHVCVTQITLKHCPDDRWTASEICGKVRMGVPPCA

>AgamOBP50

MDLHEMCHRGTLLLSSLLLLSFQRCLSPFLQSIKVASCCQLEAFLTLPTYGNCLQTIAEKYPDALWQGTVCAFDCTYREMGILTGVDDINVEQISTNQAGYDQAYQEAIAKAVTACMAQKDKIREEADVVQSECSMFAVKFHACVSLETMRNCPAERWDSSVLCEKVRSGVTVCPL

>AgamOBP51

MCHRVLSLCGFLLLGLQCGWQTLAEDCMDIKIFTTKVASCCQLEEFLTLKTYGNCLNTMAEKYPNSTLDYLVCGLDCTYREMGILTGVDDINVEQISTNQAVYGEAYQEAIGKAVDACLAQRDEFREQEKFTKSECGMFALKFQGCIMVESMRNCPAERWDSSVLCEKVRSGVAVCPP

>AgamOBP52

MSTLNHLVLLLLVVSDEHSFRCPLFFSKHPKQFPPSKKQSELPYCCQTEPLIPEHVSTKCKEREAANHNPGTELFEVCYQQCIYEELEAVDGLEIRVEKLYALAEGFPADYRHAVHLAIDECVKRLRKTRHMFEQMNAQCSLFGFAVDRCVRLLIYENCPTARWSASVACTKSRQGVPFC

>AgamOBP53

MSFRSISALVILLHLFVICTPMPECISQTQKFEVPHCCQMEELIPRPSRTKCQEKAAIDHNPGFQAYFVVNCLAQCQLEELEVIDGEELHLEKLYPLTAKFPADYRHAVRQAIDECDAWLQGKKKERRRPDGKAHCPLIGMEVENCLHRTTFSNCPNSRWKASITCNKVRQGLPFC

>AgamOBP54

MDLKKSVAVVFVSFGWMMLLATAADPDCENLKNRREEMEQCCQVNMIIPLDGAEDCSSSVDETSEPHDKMMCTLECKLKSLGLLNGDDLVEAKVQEYIDRLEGDWKGTAKTIATECITTITEMKKKIQERDHDMKCSPVGAFFMMCLMKHTQAKCPEDKWQNTSFCNKMRSGECFPKRGRQ

>AgamOBP57

MGKVLILFVGALVVASVTAGRFERSVFAPRIKRDATMRCCNDGFEKSEVHAKFAEVRTACMEELGLGETTHEELIKNREHLNCITECIAKKEGIADENGALLHTDLAKVVLEHMSTIEWKVPLAEGFIQQCFDEVELTDGAFVPSDEAKCNPEGFDFVFCLWRQFTLACPEEFRDDSEKCVELRDKLTNKEDVSDLHDDIEAAE

>AgamOBP66

MQLAICVWTAVCLQRNIIEGFLTSPPVRSCGETFNLTDPRTCCSIPYLLPADVVEPCLEIPLSPIDLAGESCRAECALNRTEMLVDGHFQLETAMQQLTNATSEDSTLTKRIQYAIGACNELFLNPTAANLTDSCTSTPRLLLDCIFADTYRNCPPQYWTASDECNQLVRTLNNCPHFLVHTDTF

>AgamOBP67

MNPVVCAFGVIFVVVTLELVVAHPGKDVLGCHNGTSITVDECCAIPMLANKTVIEKCKAAHPFKPPQNTDDKGPRGHPGECIAECIMKGMGALKNEKVDGPAFRKAIEPVVKANPAFAKLLDDTVKQCHESINVDSEFTRYVTKPVCKADAKAFINCVYGTLFEQCPTNVWTQKDGCTQLKDKIKKGCAYFALRKHGGRRMRPT

>AgamOBP68

MATTIARIGSANWAKLLVLLWLVQLATAGEPNPACKTLPTVDKDNEDKCCDVPEMFPNETLNACMEEHQQSSKPPLQKSCEITTCVLKKQSLIKSDNTVDKDKIKSYIKEMVKGSDEWKTLVEKAVLEECLPLMDKDPSNVLSKLKSSLGDCDPAPALTIACAAAKFYVNCPAKDRTKSPMCDEWRTFLSKCSNSLEDLNAIFMVLENQKTR

>AgamOBP61

MNRLVCAFGVIFVVATLELVLAHPGKDVLGCHNGTSITVDECCAIPMLANKTVIEKCKAAHPFKPPQNTDDKGPRGHPGECLAECIMKGMGALKNEKVDGPAFRKAIEPVVKANPAFAKLLDDTVKQCHESINVDSEFTRYVTKPVCKADAKAFINCVYGTLFEQCPTNVWTQKDGCTQLKDKIKKGCAYFALRKHGGRRMRPT

>AgamOBP62

MATTIARIGSANWAKVLVLLWLVQLATAGEPNPACKTMPTVDKDNEDKCCDVPEMFPNETLNACMEEYQKSSKPPLQKSCEITTCVLKKQSLIKSDNTVDKDKIKSYIKEMVKGSDEWKTLVEKAVLEECLPLMDKDPSNVLSKLKSSLGDCDPAPALTIACAAAKFYVNCPAKDRTKSPMCDEWRTFLSKCSNSLEDLNAIFMVLENQKTR

>AgamOBP63

MLPCSLIVVAVIISCNLSQRLVKYRECCSMPRLLPEQVIETCRARPLPSVIPGVPDPLPENCIAECALNETGILFNGQFRVEQAVKALSTQVPNDTLTWQHVIEVASKKCYIITVGDSFYLRDVAKNLISPQCIPSSFRFLQCTFSIVYRDCPDIYWNYQNDRCGQFVVALNNCHYLFRHIWDI

>AgamOBP64

MLSFVFLASIIVGLVSSQPPAPDASCFQPTAVTAEDCCKIPKPIDNAIMEKCRAENPKPGQMPAPGVPRTEGCCIVQCAMMETGGFVNNALNTDAIKRSMASTLGADSNFGSLVNGAVDTCARQIQNDPAYSVAPISSSPDRAGCSFIPQGFVNCLYTALFKSCPAATWTESSDCQALKTKLDSGCPFFLLMGRGPRN

>AfraOBP50e

MEFTYALVVLSACLLSSAHAAAFNCSEPPNLGAFDIHSCCRMPEINLGNAPAKCSAYIKELTTQMANMNGVGGSKQTENNKNMNNQNNPDAAAVEYPAYAHVCYAECIYRETGSMVENEFNMDNVEKFLNKSVSKGDKDIVPQIVRSFEACLNNIKGHLEAVGIKTHAKLPMGCSPFASLMFSCVNAETFLNCPAKMWKNDHNCNVAKSFAAQCNPLPHVPLPTANWLKP

>AfraOBP47b

MLALKFLVSCVFLIVRYSCAEAVEIDCQRPPRLVDPSLCCKDGGRDRTTETCAQRMGIGGQHNNGPPTVEKATCFAECILKETQYMQVPEKLNYDAMRAHLQNKFSNDSAHVETMMQAYRKCEPVVQQKLQVFKQTPLGGGAAALRRGCSPYSGMLLGCSYMEYFKNCPAHRWTANEQCALAKQFVTQCGLGA

>AfraOBP49a

MKATVIFFVLICVFGRYASADDASVDCTKPPRHIPPHMCCPVPDLSTDELMKQCDQYAGPPPTPPRGAPQRQPHPHPHGPHPNPCLIECIFNKTEVMEASGELNQDKFKELLATAVKDNEEMAAVMEESFQTCIEKATEMKTKIADKISKDPEFAEKVANHRLHSPCAPFSAMVMGCIKMETFQNCPTSTWNNTEECNTMRSFMKQCKRDRATE

>AfraOBP50a

MKFLVSLLLLAALLGVNAYEFDDSTFNEYLLKELQSLQVDDTGDALPTHRARRETEVEHSAEKDAKECAKHTWKKDMHCCKGSNVNGEQLELFMNVKKECVAELKGEPADDAYDPFNCDKMQQVKEQMICVAECVAKKFHSIDENGQFKRDVILEQLGKQIGDVQWKKDALEGYVDKCLAEVKEKHEQLEKAGKLSEGCSRCPLAFSGCMWREFWNGCPAELHVDTPKCNKLRERVAKNDTKFFGKYLLYIYYPNSDE

>AoblOBP50a

MKFLVSLLLLAALLGVNAYEFDDSTFNEYLLKELQSLQVDDTGDALPTHRARRETEVEHSAEKDAKECAKHTWKKDMHCCKGSNVNGEQLELFMNVKKECVAELKGEPADDAYDPFNCDKMQQVKEQMICVAECVAKKFHSIDENGQFKRDVILEQLGKQIGDVQWKKDALEGYVDKCLAEVKEKHEQLEKAGKLSEGCSRCPLAFSGCMWREFWNGCPAELHVDTPKCNKLRERVAKNDTKFFGKYLLYIYYPNSDE

>AoblOBP50e

MEFTYALVVLSACLLSYAHAAAFNCSEPPNLGAFDIHSCCRMPEINLGDAPAKCSAYIKELTTQMANMNGVGGSKQTENNKNMNNQNNPDAAAVEYPAYAHVCYAECIYRETGSMVENEFNMDNVEKFLNKSVSKGDKDIVPQIVRSFEACLNNIKGHLEAVGIKTHAKLPMGCSPFASLMFSCVNAETFLNCPAKMWKNDHNCNVAKSFAAQCNPLPHVPLPTANWLKP

>AoblOBP49a-2

MLRTITLSTVWLLAAICRAEQKTTTDCSKLPKSIAPQSCCRFPEPFQNPILDECYNLHSDIGQCFVECLFNRSGICRQGKCSYQRAIDYLEREIALQQSAFKDIYKRAFKKCIAKANDVLGNIVKRFSRHGCHPLPEIIRFCVRNEMFTSCPKSYWNDKVAGCSKKRDFIRNCIKDN

>AoblOBP49a-1

MKATVIFFVLICVFGRYASADDASVDCTKHPRHIPPHMCCPVPDLSTDELMKQCDQYAGPPPTPPRGAPPRQPHPHPHGPHPNPCLIECIFNKTEVMEESGELNQDKFKELLATAVKDNEEMAAVMEESFQTCIEKATEMKTKVADKISKDPEFAEKVANHRLHSPCSPFSAMLMGCIKMETFQNCPTSAWNDTEECNTMRSFMKQCKRDRSTE

>AoblOBP47b

MLALKFLVSCVFLIVSYSCAEAVEIDCQRPPRLVDPSLCCKDGGRDQTTEACAQRMGIGGQHNNGPPTVEKATCFAECILKETQYMQVPEKLNYDAMRAHLQNKFSNDSAYVETMMQAYRKCEPVVQQKLQVFKQTPLRGGAAALRRGCSPYSGMLLGCSYMEYFKNCPAHRWTDNEQCALAKQFVTQCSLGA

**Table S3** Protein names and sequences of the 51 CSPs used in Figure 3.

>DmelCSP1

MKASLALVFCVCVGLAAAAPEKTYTNKYDSVNVDEVLGNNRVLGNYLKCLMDKGPCTAEGRELKRLLPDALHSDCSKCTEVQRKNSQKVINYLRANKAGEWKLLLNKYDPQGIYRAKHEGH

>DmelCSP2

MKMILALVVLGLVLVAAEDKYTTKYDNIDVDEILKSDRLFGNYFKCLVDNGKCTPEGRELKKSLPDALKTECSKCSEKQRQNTDKVIRYIIENKPEEWKQLQAKYDPDEIYIKRYRATAEASGIKV

>DmelCSP3

MGQPGFRRAIGHVSLVVALMCTTCFQVEGLPHPPATSPSPMMERMVEQAYDDKFDNVDLDEILNQERLLINYIKCLEGTGPCTPDAKMLKEILPDAIQTDCTKCTEKQRYGAEKVTRHLIDNRPTDWERLEKIYDPEGTYRIKYQEMKSKANEEP

>DmelCSP4

MLLLNKNRVISLVVNFIFLIILISSSVQADERNINKLLNNQVVVSRQIMCILGKSECDQLGLQLKAALPEVITRKCRNCSPQQAQKAQKLTTFLQTRYPDVWAMLLRKYDSA

>EbalCSP2

MKVLIVAMLAVTIAIVAAEEKYTTKYDSIDIEEILKSDRLFKNYFNCLMESGKCTPDGRELKRVLPDALKTGCAKCSEKQRAGTERVLKFIIENKPEQWKALQAKYDPDGVYFKKYENEAKQLGVKA

>EbalCSP3

MQAIAIACSVLFVIVDFTQAQDDISNSLYTTRYERLDIDTILASPRLVTNYVECLLNKKPCAPEGKALKRILPEALRTKCGRCHPSQKEVALKVITALYFDYPQYYKALQERWDPSGDYNKRFEEYLRDQKFNSIGGGDSENGEQAQVNPPQVHKKLNRNKIKTTASSSTSTTRQPFTYSNQPLSLRLISFPDNGSLLLRPLSRILANIPFFG

>EbalCSP4

MQAIAIACSVLFVIVDFTQAQDDISNSLYTTRYERLDIDTILASPRLVTNYVECLLNKKPCAPEGKALKRILPEALRTKCGRCHPSQKEVALKVITALYFDYPQYYKALQERWDPSGDYNKRFEEYLRDQKFNSIGGGDSENGEQAQRPTRQGGNNQTQGQEAKPSAAPSTDDKELPAILNRFGADDEDGYDYEKPTVKTTTQAPSAPAPQKPSAQQPQKPQQQPSATSAPSRRPSSNNNGSNSPSSGGSSKPTQNTSGGSNPTHTWTHNNHPTPSTTTTYYLKTQNPVLNIINRITQKFANTAEFIAGMLRGAVPQPQRS

>EbalCSP5

MSIKMPSIMVWAGTMSILLLMSSAAAIRSDDKNITRLLNNQVIVSRQIMCVLEKSPCDQLGRQLKAALPEVILRNCRNCSPQQAQNAQKLTTFLQTRYPDVWAMLIRKYRGV

>EbalCSP6

MFGRTIIFGLFLMTGLVQSHPTTNSAEATTPRETYDTKFDNIDIDEILGSERLLKNYVNCLAREGPCTPDGKMLQEILPDAVSTDCVKCSPKQKIGSERVTHYLIDNKPQDWERLERLYDPQGSYRKAYLEQKAEGTKESEK

>McorCSP1

MKVLIVAMLALTIAIVAGEDKYTTKYDNIDIEEILKSDRLFKNYFNCLMESGKCTPDGRELKRVLPDALKTGCSKCSDKQRAGTERVLKFIIENKPEQWKALQEKYDPDGVYFKKYENEAKQLGVKA

>McorCSP2

MFNRVIIFSLFLLTGLIQAHPTPNPAAEATTARETYDTKFDNIDIDEILGSERLLKNYVNCLAREGPCTTDGKMLQEILPDAVSTDCMKCTEKQKTGSEKVTHYLIDNKPQDWERLEKLYDPQGSYRKAYLEEKGEGTKE

>McorCSP3

MKFTLGLCILACALAIASAASTYTTKFDNFDLDMVLNNKRVLANYIKCLNDKGPCTNEGRELKKLLPDALASDCSKCTETQKKNADKVVSHFRKNLPADFDSLLKKYDPKGVY

>McorCSP4

MSIKMPSIMVWAGTMSLLLIMSSAAAIRSDDKNITRLLNNQVIVSRQIMCVLEKSPCDQLGRQLKAALPEVILRNCRNCSPQQAQNAQKLTTFLQTRYPDVWAMLIRKYKGV

>McorCSP5

MQAVAIACSVLFVIVDFTQAQDDISNTLYTKRYERLDIDTILASPRLVTNYVECLLNKKPCAPEGKALKRILPEALRTKCGRCQPSQKEIALKVITALYFDYPQYYKALREKWDPTGEYNNRFEEYLRDQKFNAIGGGIESEAGEQAQVNAQIEKKNQTKIQKSPTSTTTTTTRPPSSSFFNNNFSNQPLSLRFMSMPDSGPLLLRPLSRILANIPFFG

>McorCSP6

MQAVAIACSVLFVIVDFTQAQDDISNTLYTKRYERLDIDTILASPRLVTNYVECLLNKKPCAPEGKALKRILPEALRTKCGRCQPSQKEIALKVITALYFDYPQYYKALREKWDPTGEYNNRFEEYLRDQKFNAIGGGIESEAGEQAQGSSYSINITQRPTRQGAGQGGVQADELKPSATTNNDEKNLPEILNRFGNDEDEGGYGYEKPEMKPSSSQAPPVAAALQPSKPQKTPSQQPQSTTQKPQQPAALPSVTSAPSRRPVIIEMDETSNANKRQVVETIRRPRLRAAILPTIFAIKELKIRHFVQPIFQG

>McorCSP7

MQAVAIACSVLFVIVDFTQAQDDISNTLYTKRYERLDIDTILASPRLVTNYVECLLNKKPCAPEGKALKRILPEALRTKCGRCQPSQKEIALKVITALYFDYPQYYKALREKWDPTGEYNNRFEEYLRDQKFNAIGGGIESEAGEQAQGSSYSINITQRPTRQGAGQGGVQADELKPSATTNNDEKNLPEILNRFGNDEDEGGYGYEKPEMKPSSSQAPPVAAALQPSKPQKTPSQPSQGTTQKPQQPAALPSVTSAPSRRPSNNGNNSPNSGSSSKPAHSNNVSGSGSSSASVPLPSQTQTWNHQNHQNNHQQPAVTTTYYLNTQNPVLNIINRITMKFANTAEFIAGMLRGAVPQPQR

>AgamCSP1

MKLFVVVALALVAAVAAQDKYTSKYDNINVDEILKSDRLFGNYYKCLLDQGRCTPDGNELKRILPDALQTNCEKCSEKQRDGAIKVINYLIQNRKDQWDVLQKKFDPENKYLEKYRGQAQKEGIKLD

>AgamCSP2

MKLFVAIAFALLALAAAQEQYTTKYDGIDLDEILKSDRLFNNYFKCLMDEGRCTPDGNELKKILPEALQTNCEKCSEKQRSGAIKVINYVIENRKEQWDALQKKYDPENLYVEKYREEAKKEGIKLE

>AgamCSP3

MKFFVVVALALVAAVAAQDKYTTKYDGVDLDEILKSDRLFNNYYKCLMDTGRCTPDGNELKRILPDALKTDCAKCSEKQKSGTEKVINYLIDNRKDQWENLQKKYDPENIYVNKYREDAKKKGINL

>AgamCSP4

MERFLLLLLFVAIVLGETANETYVTKYDNIDLEEIFSSKRLMDNYMNCLKNVGPCTPDGRELKDNLPDALMSDCVKCSEKQRIGSDKVIKFIVANRPDDFAILEQLYDPTGEYRRKYMQSDALAEHVKQEDRDLSSSGDGDADTETEAHATEHNSQDHDHREGQSDAE

>AgamCSP5

MRKVWLLASVVLAFLDFVKSQEVARTLYSTRYDNLDIDTILASNRLVTNYVDCLLSRKPCPPEGKDLKRILPEALRTKCARCSPIQKENALKIITRLYYDYPDQYRALRERWDPSGEYHRRFEEYLRGLQFNQIGGSNGGSGVGNTVLSNL

>AgamCSP6

MKHLTMVAIFAMVVVLASAQKYTDKFDNIDVDRVLSNDRILNNYLKCLLDKGPCTQEGRELKKTLPDALKTNCEKCSEKQRTSSRKVIAHLEERKPQEWKKLLDKYDPEGIYKSKFEKINKRS

>AgamCSP7

MLSAAVIVVMAALVIVGPQPAAANDSQNINRLLNNQVIVSRQIMCVLEKSPCDQLGRQLKAALPEVIQRNCRNCSPQQAQNAQKLTNFLQTRYPEVWAMLIRKYGAV

>AgamCSP8

MLHNLFLSLSLYVSVCGDPSGSTCAAEATTARTQVSDEALDKALSDKRYLMRQLKCALGEVACDPVGKRLKSLAPFVLRGACPQCTPAEMNQIKKTLAHLQRNFPSEWNKLVQTYAG

>CstyCSP4

MSHLIWLGVLFALVVIIQAVPHPPATTAAPLKQTYDNKFDNIDLDEILGQERLLNNYIKCLEGLGPCTPD

GKMLKETLPDAIMTNCAKCTEHQKYGSDKVTHHLIDNRPKDWERLEKIYDPEGSYRKAYLMQKENTTTKD

DNASKD

>CstyCSP3

MKLAIVVVAFMAIAAVSAEEKYTTKFDNIDVDEILKSDRLFTNYYKCLTDEGKCTPDGRELKKTLPDALQ

TGCSKCSDKQRQNSDKVVRFIIDNKPEEWKVLEQKYDPDHVYYNKYKDEAAARGIKI

>CstyCSP1

MKTTFACLLVAVCFALVAAEKTYTNKFDNVDVDSVLGNNRILTNYIKCLMEKGPCTPEGRELKKLLPDAL

QSDCSKCTDVQKKNSQKVINFLRANRPGEWKLLLNKYDPNGSYRAKYEKQG

>AfunCSP1

MKFFVVVALALVAVVAAQDKYTTKYDGVDLDEILKSDRLFNNYYKCLLDQGRCTPDGNELKRILPDALKTDCAKCSEKQKTGTEKVINYLIDNRKDQWENLQKKYDPENIYVNMYRDEAKKKGINP

>AfunCSP2

MKLFVAIAFALLAVVVAQEQYTTKYDGIDLDEILKSDRLFNNYYKCLMDEGRCTPDGNELKKILPEALQTNCEKCSEKQRSGAIKVINYVIENRKDRWDALQKKYDPENLYIEKYREEAKKEGIKLE

>OnigCSP1

LVLLAAVATAVAAPQKYTTKYDNINLDQVLRNDRLLTNYFKCLMDQGSCTPDGEELKSHIRDALETACSKCSDKQRAGTEKAVRFLIKNKPTEFADLEKKYDPRGVYRAKYQAEAKKKGITV

>OnigCSP2

MYTTKYDGVDVDNILKNDRLLTNYFNCIMDKGKCSPDGEELKKHIPEALENMCAQCSEKQKKGGEKVLRYIFEKKQDMFRQLEAKYDPDGKYRAKYRKLAAENGYKI

>OnigCSP3

MHTGTLLVAVALVAAASAASYTNKFDNIDVDRILSNDRILTQYIKCLMEEGSCTNEGRELKKTLPDALRTGCSKCTEKQKAATDKVIRHLRKNRSQDWNRLVTKYDPSGEFRNKYEDRLSGATR

>BdorCSP1

MKAAIIFLIVVAVQYAAAQKQYTNKFDNVDVDGVLSNNRILTNYIKCLMDKGPCTPEGRELKKLLPDALQ

TDCSKCTDTQRKNSQKVINFLRVNRPGEWKLLLDKYDSRGVYRSKYEKQG

>BdorCSP2

MKLFILAGVLALAYFTTAEDKYTTKYDNIDVDEILKSDRLFNNYFKCLIETGKCTPEGRELKKTLPDALK

TECSKCSEKQKQNTDKVIRYVIDNKPDEWKQLQAKYDPEGIYAAKYKKEAEKQGITI

>BdorCSP3

MLRFIAASVLICVVYHVATTSAAPHPPTTAAPLVANQAAYDTKFDNIDLDEVLNQERLLRNYIKCLENTG

PCTPDSKMLKEILPDAISTDCAKCSEKQRLGSAKVTHFLIDNRPEDWARLEQIYDPQGNYRLNYLAAKDK

GDGVEKTTETVTKTQA

>BdorCSP4

MKITNVTKDIGFKCIFLLLVICTCETDSDDKNINKLLNNQVIVSRQIMCVLEKSPCDQLGRQLKAALPEV

ILRNCRNCSPQQAQNAQKLTNFLQARYPDVWAMLLKKYQNI

**Table S4**. Primers used in RT–qPCR for quantifying expression levels of OBP and CSP genes from *E. balteatus*.

| Gene | Primer sequence (5'-3') | Amplicon size  (bp) | PCR efficiency  (%) | Correlation coefficients  (R^2^) |
| --- | --- | --- | --- | --- |
| CSP1 | TGTGGATATGGTTCTAGCC | 116 | 95% | 0.9953 |
|  | AAGCATCAGGAAGGAGTT |  |  |  |
| CSP2 | ACGATAGCATTGATATTGAAGAA | 103 | 100% | 0.9994 |
|  | TCACGACCATCTGGAGTA |  |  |  |
| CSP3 | GGAGAACAAGCACAGGTA | 137 | 100% | 0.9998 |
|  | AGTCGCAGTGATAATGGT |  |  |  |
| CSP4 | CACATAATAATCATCCAACTCCTA | 143 | 99% | 0.9815 |
|  | CACTGCTCCTCTTAACATTC |  |  |  |
| CSP5 | TCAGCAGGCAGATAATGT | 111 | 101% | 0.9985 |
|  | GCGAACAATTACGGCAAT |  |  |  |
| CSP6 | TGAAATGCTCACCGAAAC | 141 | 98% | 0.9992 |
|  | CCTTCTGTTCCAAGTATGC |  |  |  |
| OBP1 | TGTGCTGCTGAATGTATTG | 144 | 92% | 0.9953 |
|  | TCCATCAAGATATTCCTCCAA |  |  |  |
| OBP2 | ATTCTACACCGATACTATTCAAG | 136 | 94% | 0.9994 |
|  | ATCCTGCCAATCTTCACA |  |  |  |
| OBP3 | TCATCCACATCCATCACC | 123 | 96% | 0.9999 |
|  | CATGCTTCATCATTCTTCCA |  |  |  |
| OBP4 | AATGGATCACTACGGAAGAA | 114 | 95% | 0.9981 |
|  | AATGGTGGTGGACGATTA |  |  |  |
| OBP5 | ATTGTGCTGATGATAATAGTGAA | 120 | 98% | 0.9829 |
|  | GTTGTATAAATCTGGCTTTCCT |  |  |  |
| OBP6 | CAATACCCTGAACATTAAACAAG | 131 | 90% | 0.9959 |
|  | CCTCTACTGTCGCTGAAT |  |  |  |
| OBP7 | GGAGTGATACTGAGGGATTT | 145 | 101% | 0.9961 |
|  | TCTAATGGTGTGCTTCCTT |  |  |  |
| OBP8 | GCTATCCAGTATCACCAGAA | 121 | 107% | 0.9972 |
|  | CCCTCAGTATCACTCCAAA |  |  |  |
| OBP9 | ACAATGGACGAATGGAGT | 107 | 94% | 0.9996 |
|  | ACTTCTTTGGGTGGGTATT |  |  |  |
| OBP10 | AAGATGGCATAATTCAAGAAGA | 115 | 100% | 0.9946 |
|  | GTATCACAATTACTCGTTCCA |  |  |  |
| OBP11 | TGTGCTGATAACAATAGTGAAG | 116 | 98% | 0.9951 |
|  | GCTCCAAATCTGCCTTTC |  |  |  |
| OBP12 | ATGCTACAACTCACTGCTA | 108 | 102% | 0.9959 |
|  | GTCCACCAACCATTTGATT |  |  |  |
| OBP13 | TACTTCCCGATGAAACTGATA | 142 | 101% | 0.9976 |
|  | ACTTCCACAGAATCTCCAG |  |  |  |
| OBP14 | TTGTGGACTGGAATGGAT | 134 | 99% | 0.9817 |
|  | TTGGCTTCCTTCTTGTTG |  |  |  |
| OBP15 | AGAATGGCAAGAAGTTGAAG | 128 | 98% | 0.9945 |
|  | CACGCATGTAACAGAGATT |  |  |  |
| OBP16 | AATGACGGCTATCCTTGAT | 102 | 92% | 0.9998 |
|  | CTTCCTTCGCTATTGTTGT |  |  |  |
| OBP17 | CGAGCAGTGTAGTGTTCT | 127 | 97% | 0.9972 |
|  | AGTTGTTCTGTAGTGACCTTA |  |  |  |
| OBP18 | GATTGTAATACCCGTTTCCAT | 136 | 95% | 0.9995 |
|  | ATCCATCTTTATCGTTCCAAAT |  |  |  |
| OBP19 | CTGAGATAGCGAATCATTGTAA | 134 | 98% | 0.9993 |
|  | ACTGTCTTCCTCCAACTC |  |  |  |
| OBP20 | GAAGAGGCAATCAAGGAATT | 149 | 96% | 0.9946 |
|  | GAATCTGGCAGCATATCG |  |  |  |
| OBP21 | CCATTGTCAGTTATCCAGTT | 132 | 95% | 0.9824 |
|  | GTCTTCATACCATCCTTCAC |  |  |  |
| OBP22 | CTTGTGGATTATGCGATGG | 107 | 97% | 0.9985 |
|  | ACTTCTGCCGAAACCTTA |  |  |  |
| OBP23 | CCTTAAATGCTACATGAATTGC | 143 | 92% | 0.9999 |
|  | GGTGAATACAGTTCTTTGACA |  |  |  |
| OBP24 | CAGAAGGAGATGCTGAGAA | 129 | 94% | 0.9919 |
|  | TCTTTGCTTCGGATAATATGC |  |  |  |
| OBP25 | CGGTCTATTCAATGCTAAGG | 123 | 102% | 0.9997 |
|  | CGTTGTTCGTTCTTGTCA |  |  |  |
| OBP26 | GAAGATGTGCCTGACGAT | 146 | 105% | 0.9996 |
|  | ATATCACCAACAAGAGGATGA |  |  |  |
| OBP27 | AGTGTTTCATAGAATGCGATAT | 101 | 109% | 0.9972 |
|  | CTTTCCAACTTTAGCGGTAA |  |  |  |
| OBP28 | AAACATTACAATTTGACAACTGG | 132 | 92% | 0.9994 |
|  | TTCCTGAGCATCTTCTTGA |  |  |  |
| OBP29 | CGTTCTGGCGAATATGATG | 142 | 105% | 0.9996 |
|  | CACCGATATGAGGAGCAA |  |  |  |
| OBP30 | GTAACTTGGCACACTATACC | 142 | 110% | 0.9964 |
|  | AATCCTTGAGCATCTTCTTG |  |  |  |
| OBP31 | TGTGCTCCAAGTGAATAATG | 133 | 94% | 0.995 |
|  | GTTGATGAGGAACTAATTGCT |  |  |  |
| OBP32 | GTGTAACCGATGAGGAGATA | 128 | 98% | 0.9997 |
|  | CAATACATCACCATCCAGAG |  |  |  |
| OBP33 | TGTTAATGCTGCCAATATCC | 110 | 96% | 0.9995 |
|  | CCTTAATCTTTGCGAGTGTT |  |  |  |
| OBP34 | CCAGGAAGATGTTGTTATTGA | 108 | 92% | 0.9983 |
|  | AAACGGAAGGCAGTATCA |  |  |  |
| OBP35 | CCAGGAAGATGTTGTTATTGA | 110 | 98% | 0.9946 |
|  | GCAAACGGAAAGCAGTAT |  |  |  |
| OBP36 | TCTTGCATTCATTGCTGTT | 123 | 96% | 0.9996 |
|  | GTGTATTCTTCATCGGTCATT |  |  |  |
| OBP38 | GGAAGCCGATGTGACTAA | 137 | 100% | 0.9954 |
|  | TTAGCAGAACCTTATCATCAAC |  |  |  |
| OBP39 | GCCAGCAAAGATGTTCAA | 150 | 94% | 0.9983 |
|  | TTGTTCACAGTCGTCTCC |  |  |  |
| OBP40 | GAAGTTGCTAATCCTGATGAA | 128 | 97% | 0.9999 |
|  | GCACCAAGTTTCTTCTCAA |  |  |  |
| OBP41 | AATCCTGGTTGCTATCTGT | 149 | 99% | 0.99 |
|  | CATTAGTCACATCAGCTTCTT |  |  |  |
| OBP42 | CTGATGTAACTCGTGATATGAA | 124 | 95% | 0.9998 |
|  | GTATCTTCGTCAGCATAATCAT |  |  |  |
| OBP43 | AACTCATGTCGTGGTGAA | 100 | 93% | 0.9919 |
|  | CCTTCTTCTGTAACAACTCAAT |  |  |  |
| OBP45 | ATGTATGAGAAGAACGGAGT | 122 | 101% | 0.9996 |
|  | TCAACCAGACCAAGAAGT |  |  |  |
| OBP49 | GGAGCCACAGAAGATCAA | 100 | 95% | 0.9964 |
|  | CAATGTTGTCAGCTACTTCA |  |  |  |
